# Antibody drug conjugate: the “biological missile” for targeted cancer therapy

**DOI:** 10.1038/s41392-022-00947-7

**Published:** 2022-03-22

**Authors:** Zhiwen Fu, Shijun Li, Sifei Han, Chen Shi, Yu Zhang

**Affiliations:** 1 Department of Pharmacy, Union Hospital, Tongji Medical College, Huazhong University of Science and Technology, Wuhan, 430022 People’s Republic of China; 2 Hubei Province Clinical Research Center for Precision Medicine for Critical Illness, Wuhan, 430022 People’s Republic of China; 3 Drug Delivery, Disposition and Dynamics, Monash Institute of Pharmaceutical Sciences, Monash University, (Parkville Campus) 381 Royal Parade,, Parkville, VIC 3052 Australia; 4 Faculty of Pharmacy, China Pharmaceutical University, 639 Longmian Avenue, Jiangning District, Nanjing, 211198 People’s Republic of China

**Keywords:** Drug development, Drug development

## Abstract

Antibody–drug conjugate (ADC) is typically composed of a monoclonal antibody (mAbs) covalently attached to a cytotoxic drug via a chemical linker. It combines both the advantages of highly specific targeting ability and highly potent killing effect to achieve accurate and efficient elimination of cancer cells, which has become one of the hotspots for the research and development of anticancer drugs. Since the first ADC, *Mylotarg*^*®*^ (gemtuzumab ozogamicin), was approved in 2000 by the US Food and Drug Administration (FDA), there have been 14 ADCs received market approval so far worldwide. Moreover, over 100 ADC candidates have been investigated in clinical stages at present. This kind of new anti-cancer drugs, known as “biological missiles”, is leading a new era of targeted cancer therapy. Herein, we conducted a review of the history and general mechanism of action of ADCs, and then briefly discussed the molecular aspects of key components of ADCs and the mechanisms by which these key factors influence the activities of ADCs. Moreover, we also reviewed the approved ADCs and other promising candidates in phase-3 clinical trials and discuss the current challenges and future perspectives for the development of next generations, which provide insights for the research and development of novel cancer therapeutics using ADCs.

## Introduction

Cancer has become the second greatest global health threat, accounting for approximately 10.0 million deaths from cancer occurred in 2020.^1^ Cytotoxic agents based chemotherapy has been the main approach for the treatment of a wide range of cancers for decades.^2^ These cytotoxic agents include analogs of DNA bases (5-fluorouracil and 8-azaguanine), DNA interacting agents (cisplatin and actinomycin D), antimetabolites (aminopterin and methotrexate), and tubulin inhibitors (paclitaxel and vincristine derivatives), *etc.*^3–7^ Most of these chemotherapy agents, however, show low therapeutic index, where severe side effects are generally attributed to non-specific drug exposure to off-target tissues.^8^ To address this issue, scientists have been working on the development of novel cancer therapeutics with higher targeting ability.

As early as the beginning of 20th century, Paul Ehrlich first proposed the concept of “magic bullets” and postulate that some compounds could directly access to some desired targets in cell to cure diseases.^9^ Theoretically, these compounds should be effective in killing cancer cells, but harmless to normal cells. One of the plausible ways is to identify some specifically overexpressed antigens to distinguish cancer cells from health cells, such as HER2 (human epidermal growth factor receptor 2) on the breast cancer and CD20 (cluster of differentiate 20) on the B cell lymphoma.^10,11^ Specific expression of these antigens provides the possibility of precision tumor targeting via monoclonal antibodies (mAbs), and this field was advanced greatly after the development of hybridoma technology since 1975.^12^ In recent decades, an increasing number of mAbs, such as avastin, trastuzumab, rituximab, and cetuximab, have been received approval worldwide for treatment of various solid tumors and hematological cancers.^13–16^

The emergency of mAbs has changed the paradigm of cancer therapy through precise targeting tumor surface antigens, however, treatment using mAbs alone is often insufficient, potentially due to less satisfactory lethality against cancer cells compared to chemotherapy.^17^ Hence, a novel concept, known as antibody–drug conjugate (ADC), was conceived to bridge the gap between the mAb and cytotoxic drug for the improvement of therapeutic window.^18^ ADC consists of a tumor targeting mAbs conjugated to a cytotoxic payload through a sophisticatedly designed chemical linker, enabling the ability of precise targeting and potent effectiveness simultaneously. Moreover, owing to the conjugation to a large hydrophilic antibody, the antigen-independent uptake of cytotoxic payload in those antigen-negative cells is limited, contributing to widening therapeutic index.^19^

**Fig. 1:**
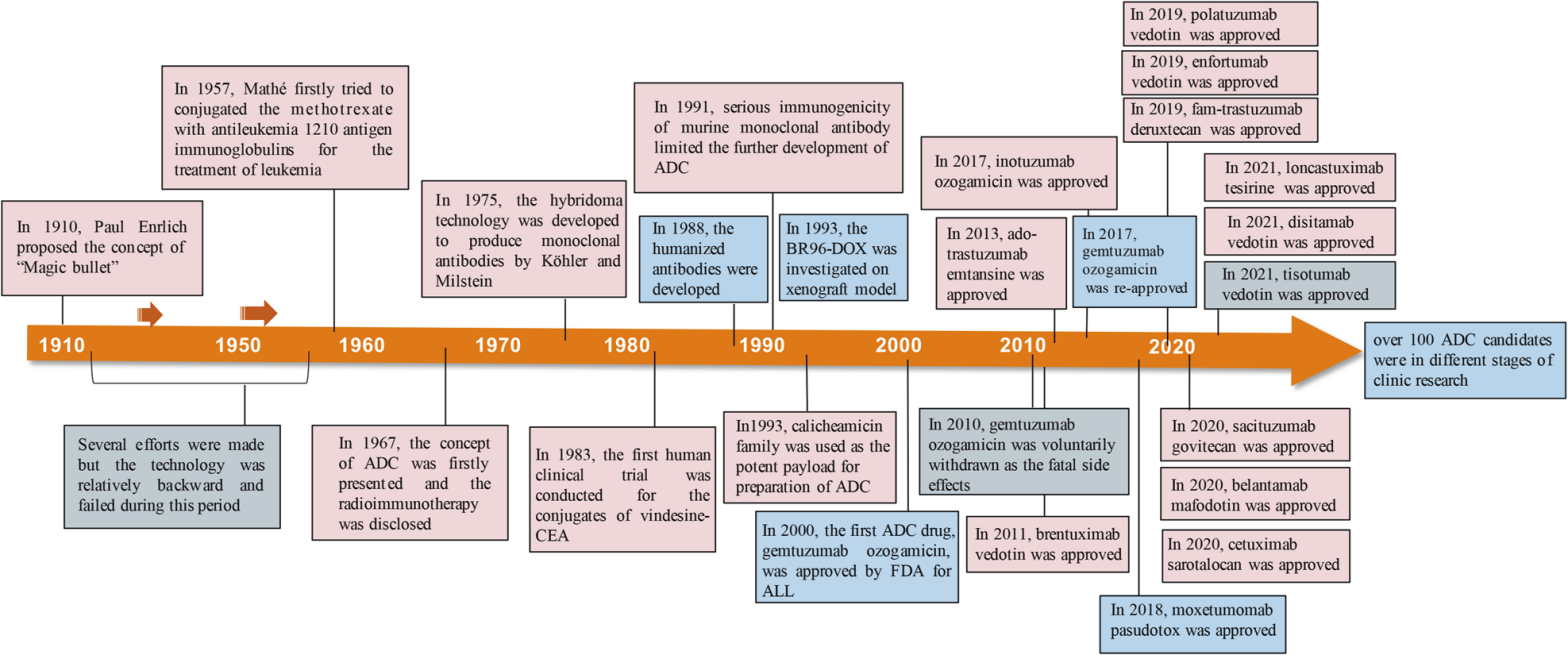
Timeline depicting important events in the development and approval of ADC drugs over the past century since the “magic bullet” was proposed by Paul Enrlich 1910. ADC, antibody-drug conjugate; CEA, Carcinoembryonic antigen; ALL, acute lymphoid leukemia; BR96, an antibody binding to Lewis Y; DOX, doxorubicin; FDA, the U.S. Food and Drug Administration

In 2000, the U.S. Food and Drug Administration (FDA) firstly approved ADC drug, *Mylotarg*^*®*^ (gemtuzumab ozogamicin), for adults with acute myeloid leukemia (AML), which marked the beginning of ADC era of cancer targeted therapy.^20^ By December 2021, there have been 14 ADC drugs approvals for both hematological malignancies and solid tumors worldwide. Moreover, over 100 ADC candidates are in the different stages of clinical trials at present. The landmark event in ADC drug from its infant stage to the mature development stage over the past hundred years was depicted in Fig. 1. With expanding targets and indications, ADC is leading a new era of targeted cancer therapy and it is expected to be a substitute for conventional chemotherapies in the future.^21^ In this review article, we provide a discussion of the molecular aspects of key components and general mechanism of action of ADC, and briefly summarized the advance in the development of ADC. We also reviewed the approved ADCs and other promising candidates in phase-3 clinical trials and discuss the current challenges and future perspectives for the development of next generations of ADC.

## Key components of ADC

**Fig. 2:**
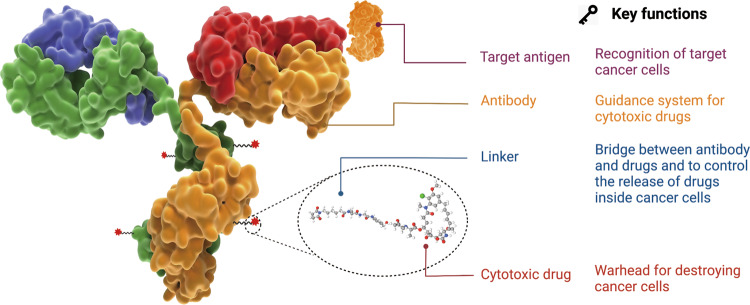
The structure and characteristic of an ADC drug. The core components including target antigen, antibody, linker, cytotoxic drug along with their key functions are demonstrated.

As shown in Fig. 2, ADC is composed of antibody, cytotoxic payload and chemical linker. An ideal ADC drug remains stable in blood circulation, reaches the therapeutic target accurately, and eventually releases the cytotoxic payloads in the vicinity of the targets (*e.g*. cancer cells). Each element can affect the final efficacy and safety of ADC, and in general ADC development needs to take into account all these key components, including the selection of target antigen, antibody, cytotoxic payload, linker, as well as conjugation methods.

### Target antigen selection

The target antigen expressed on tumor cells is the navigation direction for ADC drugs to identify tumor cells and it also determines the mechanism (e.g., endocytosis) for the delivery of cytotoxic payloads into cancer cells. Hence, an appropriate selection of target antigen is the first consideration for the designation of ADC. In order to reduce off-target toxicity, the targeted antigen firstly should be expressed exclusively or predominantly in tumor cells, but rare or low in normal tissues.^22^ The antigen is ideally a surface (or extracellular) antigen rather than an intracellular one in order to be recognized by circulating ADCs. For example, the expression of HER2 receptor in certain types of tumors is approximately 100 times higher compared to normal cells, which services as a solid foundation for the development of ado-trastuzumab emtansine, fam-trastuzumab deruxtecan and disitamab vedotin.^23^ Secondly, the target antigen should be non-secreted since secreted antigen in the circulation would cause the undesirable ADC binding outside tumor sites, resulting in the decreased tumor targeting and elevated safety concerns.^24^ Thirdly, the target antigen is ideal to be internalized upon binding with the corresponding antibody, so that the ADC-antigen complex gain access into cancer cells, followed by appropriate intracellular transport route and avid release of cytotoxic payload.^25^

**Fig. 3:**
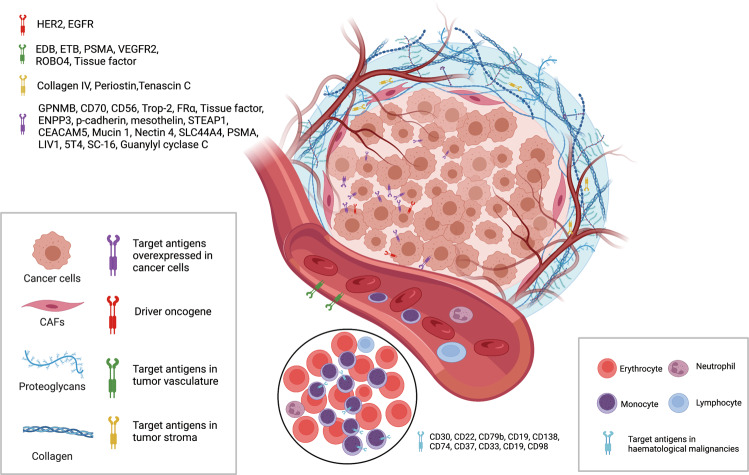
The important target antigens from tumor cells (overexpressed and driver genes) and tumor microenvironment (vasculature and stroma) are used for the development of ADC. Created with BioRender.com

At present, as shown in Fig. 3, the target antigens of the approved ADC drugs are typically specific proteins overexpressed in cancer cells, including HER2, trop2, nectin4 and EGFR in solid tumors, and CD19, CD22, CD33, CD30, BCMA and CD79b in hematological malignancies.^26^ Driven by fundamental research in oncology and immunology, the selection of ADC target antigen has gradually extended from conventional tumor cell antigens to targets in the tumor microenvironment, *e.g*. in the stroma and vasculature. Emerging evidence in the preclinical and clinical setting suggests that components of the neovascular system, subendothelial extracellular matrix and tumor matrix could be valuable target antigens for ADC drug development.^27^ For example, matrix targeted ADC drugs has the potential to cause cancer cell death by reducing the concentration of growth factors produced by matrix-resident cells. Since the survival of cancer cells depends on angiogenesis and matrix factors, ADCs may have a broader efficacy by targeting such tissues. Moreover, the genome of these cells is more stable than that of cancer cells, which could provide a promising mean to reduce the possibility of mutation induced drug resistance.^28^

### Antibody moiety

The tumor targeting antibody is critical for specific binding between the target antigens and the ADC. In addition to high binding affinity to the target antigen, an ideal antibody moiety should also facilitate efficient internalization, demonstrate low immunogenicity and preserve long plasma half-life.^29^ At the early stage of the development of ADC drugs, mouse-derived antibodies were predominantly employed, where high failure rates were observed due to serious immunogenicity-related side effects.^30^ With the emergence of recombinant technology, murine antibodies was mostly replaced with chimeric antibodies and humanized antibodies.^31^ At present, ADCs increasingly employed fully humanized antibodies with significantly reduced immunogenicity. Among 14 approved ADC drugs, only brentuximab vedotin uses chimeric antibody.

As the main component of immunoglobulin in serum, the antibodies currently used in ADC drugs are mostly immunoglobulin G (IgG) antibody, which includes four subtypes, namely IgG1, IgG2, IgG3 and IgG4. IgG1 the commonly used subtype for ADCs as IgG1 is the most abundant in serum and could induce the strong effector functions such as antibody-dependent cell-mediated cytotoxicity (ADCC), antibody dependent phagocytosis (ADCP), and complement dependent cytotoxicity (CDC) by a high binding affinity with Fc receptor.^32^ These Fc-mediated effector functions play crucial roles in anticancer activity of antibody drugs. IgG3 is rarely employed in ADC because of the rapid clearance rate. Unlike the other three subtypes with half-lives of approximately 21 days, the half-life of IgG3 is only approximately 7 days in serum.^33^ IgG2 often shows tendency to form dimers and aggregations in vivo, which leads to a decrease of the concentration of ADC drugs.^34^ IgG4 could induce ADCP, however, IgG4 is an unusually dynamic antibody with Fab-arm exchange, resulting in the reduced efficacy and ineffective targeting effect.^35,36^

With regards to internalization of the antibody-antigen complex, the efficiency mainly depends on the binding affinity between the antibody and the surface antigen on the tumor cells, where higher affinity often results in more rapid internalization.^37^ However, antibodies with high antigen affinity may in turn reduce the penetration into solid tumors. The treatment of solid tumors is more complex than blood tumors because of the existence of “binding site barrier (BSB)” in solid tumors,^38^ where extremely strong binding between the antibody and the antigen results in trapping of ADCs near the blood vessels after they extravasate but less penetration to tumor cells away from the blood vessels.^39^ Hence, a reasonable affinity between antigen and antibody should be optimized to balance the rapid absorption in the target cells and anticancer potency. In addition to binding affinity, another factor that influences tumor penetration is the size of the antibody. The large molecular weights of IgG antibodies (approx. 150 kDa) often presents a challenge for penetration through the blood capillary and the matrix in tumor tissues.^37^ Early ADCs hence mainly target hematological malignancies. In order to make ADCs better applicable to solid tumor treatment, researchers have tried to miniaturized the antibodies by removing the FC segment. The miniaturized antibodies not only retain high affinity and specificity, but also penetrate through blood vessels into solid tumors more easily, thereby greatly improving the killing effect on solid tumors. However, such changes have also been found to cause the reduction of half-life in vivo.^40^ Therefore, various factors should be considered when designing ADCs with miniaturized antibodies.

### Linkers

Linker in ADC bridges the antibody with the cytotoxic drug. It is one of the key factors related to the stability of ADC and payload release profiles, and is therefore important for the ultimate therapeutic index of ADCs. An ideal linker should not induce ADC aggregation, and it is expected to limit premature release of payloads in plasma and to promote active drugs release at desired targeted sites. Depending on the metabolic fate in cells, two types of linkers including cleavable and non-cleavable linkers have been employed in most of ADC drugs.

Cleavable linkers take advantage of the environmental differences between the systemic circulation and tumor cells to accurately release of the free cytotoxic drugs, and they can be further categorized into chemical cleavage linkers (hydrazone bond and disulfide bond) and enzyme cleavage linkers (glucuronide bond and peptide bond).^41^ Hydrazone is a typical acid-sensitive (pH sensitive) linker. Hydrazone linked ADCs are generally stable in blood circulation but hydrolyzed to release the cytotoxic payloads in lysosome (pH 4.8) and endosome (pH5.5–6.2) upon internalization into the targeted cancer cells.^42^ However, hydrolysis of the hydrazine bond is not completely confined to the lysosomes, and occasional hydrolysis also occurs in the plasma, resulting in reduction of targeting efficiency and off-target effects.^43^ So far, hydrazine linker containing ADCs are mainly used in hematological malignancies. For example, gemtuzumab ozogamicin and inotuzumab ozogamicin both use the hydrazone to link calicheamicin with mAbs for the treatment of AML and acute lymphoblastic leukemia (ALL), respectively. Disulfide bond based linker is another chemically sensitive cleavable linker that is sensitive to reductive glutathione (GSH).^44^ GSH plays a crucial role during cell survival, cell proliferation and differentiation for the maintenance of the intracellular redox balance.^45^ The concentration of GSH in blood is considerably lower than intracellular concentration in cancer cells.^46^ Therefore, this type of linker could keep stable in blood system while specifically release the active payloads in the cancer cells with an elevated GSH level.

In terms of enzyme sensitive linkers, peptide based linker is sensitive to the lysosomal protease and have been employed in a number of ADCs.^47^ The lysosomal proteases, such as cathepsin B, are generally overexpressed in cancer cells, enabling the accurate drug release in the vicinity of the tumor.^48^ Moreover, because of the existence of protease inhibitors in the blood, the linker are normally stable in the systemic circulation and it decreases the risk of side effects.^49^ Among approved ADC drugs, 9 of 14 use peptide based linkers. For example, brentuximab vedotin uses a valine-citrulline linker. Besides, beta-glucuronide linker is another enzyme-sensitive linker commonly used in ADCs. It can be cleaved for payloads release in cells by beta-glucuronidase, the levels of which are often found higher in tumor regions.^50^

In contrast, non-cleavable linkers (e.g., thioether or maleimidocaproyl group) are inert to common chemical and enzymatic environments in vivo. The biggest superiority of non-cleavable linker is its low off-target toxicity benefited from an increase of plasma stability.^51,52^ The non-cleavable linker depends on the enzymatic hydrolysis of the antibody component of ADC by protease, and finally releases the payload “complex”, which is drug connected with the amino acid residue in an antibody degradation product.^53^ Only small molecules that tolerate chemical modifications (e.g., when pharmacophore is far away from the conjugation site) are suitable for thioether based linker. The ado-trastuzumab emtansine (T-DM1) demonstrates a successful application of thioether linker.^54^ The conjugate is the product of anti-HER2 monoclonal antibody linked with DM1 (mertansine) via a succinimidyl‐4‐(N‐maleimidomethyl)cyclohexane‐1‐carboxylate (SMCC) linker. The linker makes the conjugate more stable in blood and release of active metabolite of DM1, lysine-MCC-DM1, after a digestion of the antibody moiety by protease inside cancer cells.^55^

### Cytotoxic payloads

The cytotoxic payload is the warhead that exerts cytotoxicity after internalization of ADCs into cancer cells. Because only approximately 2% of ADC could reach targeted tumor sites after intravenous administration,^26^ high potency (IC_50_ in nanomolar and picomolar range) is required for the compounds to be used as payloads in ADC.^56^ Moreover, these compounds should keep stable in physiological conditions and have available function groups for conjugation with the antibody.^57^ At present, the cytotoxic payloads used for ADCs mainly include potent tubulin inhibitors, DNA damaging agents, and immunomodulators (Table 1).^23^

**Table 1:** The representative small-molecule payloads used in ADC drugs

Categories	Names	Structures	Mechanisms	Potency (IC_50_ or EC_50_)
Tubulin inhibitors	Auristatins		Promote tubulin polymerization and target at the β-subunits of tubulin dimer to perturb microtubule growth	0.05–0.1 nM
	Maytansinoids		Block the polymerization of tubulin dimer and inhibit the formation of mature microtubules	0.05–0.1 nM
	Tubulysins		Inhibit tubulin polymerization	0.1–1 nM
DNA damaging agents	Calicheamicins		DNA double strand break: bind with DNA in the minor groove and cause strand scission	0.1–1 nM
	Duocarmycins		DNA alkylation: bind to the minor groove of DNA and alkylate the nucleobase adenine at the N3 position	1–10 pM
	Exatecans		Topoisomerase I inhibitor: bind to the topoisomerase I and DNA complex and prevent DNA re-ligation and therefore causes DNA damage which results in apoptosis	1–10 nM
	Pyrrolobenzodiazepines		Crosslinking of DNA: produce DNA interstrand cross-links with high efficiency in both naked DNA and in cells.	0.1–1 pM
Immunomodulators	TLR agonists		Potent stimulation of innate and adaptive immunity as well as their effects on the tumor microenvironment	~1 μM
	STING agonists		Promote activation of type I interferons and other inflammatory cytokines	~100 nM

Microtubules are the main component of cytoskeleton and play a significant role in cell division, particularly during rapid proliferation of tumor cells.^58^ Tubulin inhibitors including tubulin polymerization promoters and tubulin polymerization inhibitors that interfere with microtubule-dependent mitosis have become one of the research and development hotspots of anticancer drugs.^59,60^ Tubulin polymerization promoters target at the β-subunits of tubulin dimer to perturb microtubule growth, and they are exemplified by auristatin derivatives monomethyl auristatin E (MMAE) and monomethyl auristatin F (MMAF).^61,62^ Among the 14 approved ADC drugs, 5 of them use MMAE/MMAF as the payloads. In contrast, the inhibitor of tubulin polymerization blocks the polymerization of tubulin dimer to form mature microtubules. Typical inhibiting agents include maytansinoid derivatives DM1 and DM4 (ravtansine).^63^ Ado-trastuzumab emtansine, approved by the FDA in 2013, was the first ADC drug conjugated using maytansinoid derivatives. In addition, the tubulysins (tubulysin A-D, tetrapeptides isolated from myxobacterial) are another class of tubulin polymerization inhibitor that show promising anticancer activity.^64^ For example, EC1169, a prostate-specific membrane antigen (PSMA) targeted conjugate of tubulysin B hydrazide, is currently under clinical trials (NCT02202447).^65^

Compared with the nanomolar range of IC_50_ (half-maximal inhibitory concentration) seen in microtubule inhibitors, the IC_50_ values of DNA damaging agents are able to reach picomolar level, thus ADCs conjugated with DNA damaging agents are sometimes more effective and may work independent to cell cycles (compared to tubulin inhibitors that work mainly on the mitocytosis phase), and they may even for those cells with a low antigens expression.^66^ The detailed mechanisms involved in DNA damaging agents mainly include: (i) DNA double strand break, such as calicheamicins;^67^ (ii) DNA alkylation, such as duocarmycins;^68^ (iii) DNA intercalation, such as topoisomerase I inhibitors;^69^ (iv) DNA crosslink, such as pyrrolobenzodiazepines (PBD).^70^ Calicheamicin is a natural enediyne antibiotics, which is extremely potent for DNA damaging.^71^ After binding with DNA in the minor groove, calicheamicin produces free radicals and causes strand scission thereby inducing cell death. Among derivatives of calicheamicin, calicheamicin γ1 is the most notable one and is used in gemtuzumab ozogamicin and inotuzumab ozogamicin. Duocarmycin is another class of exceptionally potent antitumor antibiotics that binds to the minor groove of DNA and alkylates the nucleobase adenine.^72^ SN-38 (7-ethyl-10-hydroxycamptothecin) and DXd (exatecan derivatives) are two main derivatives of camptothecin used in ADC drugs as payloads through inhibition of DNA topoisomerase I.^73,74^ For example, sacituzumab govitecan is a first-in-class Trop-2 targeting ADC that conjugates SN-38 to sacituzumab and fam-trastuzumab deruxtecan is composed of a HER2-directed antibody coupled to DXd by a peptide linker. PBD is a class of antitumor antibiotics discovered as early as 1960s. PBD works in as a dimer to bind to the DNA minor groove.^75^ After binding, the dimer facilitates amino cross-linking with guanine at N2 position of DNA and thus prevents combination of DNA and transcription factors, resulting in stagnation of cell proliferation and eventually cell death. This mechanism does not depend on a specific cell replication cycle and the DNA damage is difficult to repair, resulting in potent cytotoxicity.^76^ Loncastuximab tesirine is currently the only ADC in clinical use that employs PBD as the payload.^77^

In addition to traditional cytotoxins, an increasing number of payloads with new mechanisms are being incorporated into ADC design. For example, the small-molecule immunomodulators recently began to be applied to development of novel ADC drugs, which are also termed as immune-stimulating antibody conjugates (ISACs).^78^ ISACs combine the precision of antibody-navigated targeting and the power of small molecule based modulation of the innate and adaptive immune systems. Promising tumor regression and long-term anti-tumor immunity in a variety of tumor models have been documented.^79^ At present, novel payloads mainly include toll like receptor (TLR) agonists and stimulator of interferon genes (STING) agonists.^80,81^ TLRs are a group of crucial pattern recognition receptors in innate immunity that play important roles in the immune-tumor interface.^82^ For example, activation of TLR7 and/or TLR8 could induce MyD88 dependent signaling pathway that activate NF-κB for the secretion of cytokines and chemokines, allowing infiltration of anti-tumor lymphocytes.^82^ BDC-1001 is a Boltbody ISAC that is currently in clinical development (Phase 1/II, NCT04278144).^83^ It consists of a HER2-targeting antibody linked to a TLR7/8 agonist for the treatment of patients with HER2-positive solid tumors. Silverback Therapeutics also developed the ImmunoTAC platform and designed several ISACs using TLR8 agonists as payloads, such as SBT6050, SBT6290, and SBT8230.^84^ As for STING, it is also a well-studied innate immune pathway and STING agonist are capable of inducing anti-tumor immune activity.^85^ CRD5500 from Takeda and XMT-2056 from Mersana are two leading STING-agonist ADC programs under the clinical development.^86,87^ ISACs is a relatively new area but some candidates have successively entered clinical development, and their follow-up progress is expected.^78,88,89^

### Conjugation methods

In addition to selection of the antibody, the linker and the payload, the approach by which the small molecule moiety (i.e., linker plus payload) is connected to the antibody is also important for successful construction of ADCs. Typically, the existence of lysine and cysteine residues on antibody provides the accessible reaction sites for conjugation, and the early ADC drugs usually exploit stochastic conjugation on pre-existing lysine or cysteine residues via appropriate coupling reactions.^90^ Amide coupling is arguably the most frequently used method, where an active carboxylic acid ester (when available in the linker) is used to connect payloads to lysine residues on the antibody, as seen in gemtuzumab ozogamicin, T-DM1 and inotuzumab ozogamicin. However, an antibody usually contains approximately 80–90 lysine residues, of which 40 lysine residues are typically modifiable.^26^ Through the random coupling with lysine residues, varying numbers (0–8) of small-molecule toxins may be attached to an antibody, resulting in a wide drug-antibody ratio (DAR) distribution.^91^ In addition, as the lysine residues are distributed throughout the antibody light chain and heavy chain, coupling reaction near the antibody-antigen recognition sites may reduce ADC binding to targets.^92^

Cysteine based reaction provides another means of coupling. After reduction, the disulfide bond could transform to cysteine residues which are accessible for coupling reaction. Typically, IgG1 antibodies have both interchain disulfide bonds and intrachain disulfide bonds.^93^ The interchain disulfide bonds are exposed on the outside of the antibody, and are easy to be reduced to expose free cysteine residues, providing the available sites for conjugation of linker-payload to the antibodies.^94^ Due to the limited number of binding sites and the unique reactivity of mercaptan groups, using cysteine as the connecting site helps to reduce the heterogeneity of ADC. Depending on the reduction ratio, products with DAR of 2, 4, 6 and 8 may be generated with better homogeneity compared to products from lysine residue coupling.^95^ This is so far the most commonly used coupling method in commercial products. However, it is worth to note that opening the inter chain disulfide bond may destroy the integrity of antibody.^96^

A number of disadvantages are often associate with the stochastic conjugation based on lysine and cysteine residues. The stability of such coupling is sometimes insufficient and this causes premature payload release and thus off-target toxicity.^97,98^ Furthermore, it is difficult to guarantee payload connection to consistent sites on the antibody and it is also difficult to achieve a homogeneous DAR that are favored by quality control and clinical use. In order to reduce the heterogeneity of ADCs, several site-specific conjugation strategies have been developed in new ADCs (Table 2).

**Table 2:** The characteristics of various conjugation methods applied for ADC

Conjugation strategies	Conjugation methods	Schematic diagram	Advantages	Disadvantages
Stochastic conjugation	Lysine sites		• Rapid and convenient	• Heterogeneous with random DAR (0–8); • Reduced ADC binding affinity; • Poor therapeutic index
	Reduced cysteine sites		• A relatively homogeneous product	• The structure of antibody was broken; • Off-target toxicity as premature release of payloads
Site-specific conjugation	Engineered reactive cysteine residues		• High homogeneity; • Tunable reactivity and stability	• Genetic engineering required; • Typically limited to DAR 2
	Disulfide re-bridging		• High homogeneity; • No influence on spatial structure of antibody; • National amino acid sequence and glycosylation	• Intrachain mis-bridging; • Typically limited to DAR 4
	Unnatural amino acids		• High homogeneity; • Tunable reactivity and stability; • High efficiency of conjugation	• Genetic engineering required; • Low antibody expression yields; • Immunogenicity caused by unnatural amino acids sequence; • Aggregation as the hydrophobicity of unnatural amino acids;
	Enzyme-assisted ligation		• High homogeneity; • High efficiency of conjugation • DAR alteration possible	• Genetic engineering required for installation of recognition sequence • Immunogenicity caused by extraneous amino acids sequence
	Glycan remodeling and glycoconjugation.		• High homogeneity; • No alteration of amino acid sequence	• Glycosylation profile is important in immune recognition
	pClick technology		• Without antibody engineering or chemical/enzymatic treatments • Simple, efficient, and convenient	• More antibody-binding peptides need to be explored

Firstly, the introduction of engineered reactive cysteine residues has become a common approach for site-specific conjugation. ThioMab technology developed by Genentech employed genetic engineering technology to insert cysteine residues at specific positions of light chain V110A and heavy chain A114C of trastuzumab and then coupled to sulfhydryl group on cysteine with MMAE to synthesize site-specific anti-MUC16 ADC.^99^ The percentage of produced ADC with DAR of 2 is as high as 92.1%. In addition, ThioMab technology did not affect the immunoglobulin folding and assembly or antibody binding to the antigen. On the other hand, a main limitation of the ThioMab technology is that the thiol group introduction step may cause a wrong disulfide bond formed between the two Fabs in the antibody, which remains a challenge to be addressed.^100,101^ In addition, disulfide re-bridging conjugation has attracted attention in spite of the low conjugation efficiency and intrachain mis-bridging. Similar with the conventional cysteine conjugation, the conjugation sites are also obtained through reduction of interchain disulfide bond. Instead of stochastic coupling, disulfide re-bridging involves the reaction with cysteine-selective cross-linking reagents, such as bissulfone reagents,^102^ next-generation maleimides (NGMs),^103^ and pyridazinediones (PDs).^104^ The bis-reactive reagents enable the reconnection of the polypeptide chains of antibodies as well as the conjugation of payloads on antibodies.^105,106^ The Depending on the number of payloads attached to each linker, the ADCs with DAR of 4, 8 or 16 may be produced.^107^

Another method for site-specific conjugation is through introduction of unnatural amino acids, including N-acetyl-L-phenylalanine, azido methyl-L-phenylalanine and azido lysine.^108^ Special functional groups in these unnatural amino acids enable the site-specific conjugation. Moreover, the conjugation is controllable and quantitative to generate ADCs with homogeneous DAR, high efficacy, good stability and high safety.^109^ However, it is sometimes is difficult to produce the modified antibodies and the antibody with unnatural amino acids may induce immunogenicity.^110^The hydrophobicity of unnatural amino acids also increases the risk of antibody aggregation.^108^ Enzyme-assisted ligation is also an effective strategy for site-specific conjugation.^111^ Through genetic engineering, specific amino acid sequences are artificially induced to express in the antibody and these sequences can be recognized by certain enzymes and subsequently specific amino acid residues are modified by the enzyme, so as to enable site-specific conjugation. At present, formyl glycine-generating enzyme (FGE) and transglutaminase (TG) are commonly used.^112^ However, it is worth noting that the immunogenicity may be induced upon modification of the amino acid sequences.

Site‐specific ADCs can also be generated from glycan remodeling and glycoconjugation.^113^ In the Fc fragment of antibodies, the existence of N-glycan at the N297 position of CH_2_ domain of each heavy chain enables the reactive sites for conjugation with payloads through glycosylation.^114^ The long-distance localization between the polysaccharide and the Fab region can minimize the impairment of antigen binding affinity. It may be a deficiency in the construction of ADC through lysine-based chemical conjugation.^115^ Moreover, a pClick technology was recently developed for site-specific conjugation in ADC.^116^ By introduction of a proximity-activated crosslinker, the peptide modified with azide group could be spontaneously reacted with the closest lysine residues on the antibody. And the azide groups provide the available sites for click chemistry with a bioorthogonal handle modified payload. The yield and antibody stability are hence significantly improved due to no requirement of antibody engineering and post-reaction treatment. The pClick technology provides a new option to perform site-specific conjugation for the ADC development in a more convenient and efficient way.

## Mechanism of action of ADC

**Fig. 4:**
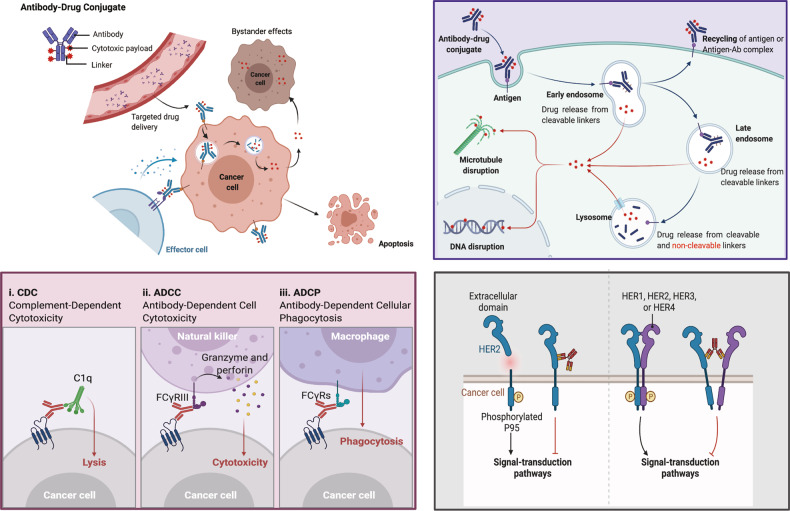
The overview of the mechanisms of ADC for killing cancer cells via different approaches. **Upper-Right:** The main core mechanism of action of ADCs; **Lower-Left:** The antibody component of ADCs engages with immune effector cells to elicit antitumor immunity including CDC, ADCC, and ADCP effects; **Lower-Right:** The antibody component of ADCs retains its activity profile and can therefore interfere with target function, dampen downstream signaling to inhibit tumor growth. Created with BioRender.com

ADCs synergistically play the “specific” targeting role and the “efficient” killing effect on cancer cells. Such drugs are like a precision guided “biological missile” with the ability to destroy cancer cells accurately, improving the therapeutic window and reducing the off-targeted side effects.^117^ A primary mechanism of action of ADC is shown in the upper-right panel of Fig. 4. Once mAb of ADC is bound to the target antigens that specifically expressed on the cancer cells, the ADC is endocytosed/internalized by cells to form an early endosome, followed a maturation into late endosomes and finally fused with lysosomes. The cytotoxic payloads are eventually via either chemical or enzyme mediated release in the lysosomes, resulting in cell apoptosis or death via targeting DNA or microtubules.^57^ When the payload released is permeable or transmembrane, it may also induce bystander effect to enhance the efficacy of ADC. Moreover, the bystander effect of these drugs may also alter the tumor microenvironment, which in turn may further enhance the killing effect of ADCs.^118^

In addition, the anticancer activity of ADC also involved in the ADCC, ADCP and CDC effects.^119,120^ The Fab segment of antibody of some ADCs could bind to the antigen epitope of virus infected cells or tumor cells while the FC segment binds to FCR on the surface of killer cells (NK cells, macrophages, etc.), thereby mediating the direct killing effects (the lower-left panel of Fig. 4). Furthermore, the antibody component of ADC could specifically bind to the epitope antigen of cancer cells and inhibits the downstream signal transduction of antigen receptor (the lower-right of Fig. 4). For example, the trastuzumab of T-DM1 can bind to the HER2 receptor of cancer cells and block the formation of heterodimer between HER2 and HER1, HER3 or HER4, which inhibits the signal transduction pathways (like PI3K or MAPK) of cell survival and proliferation to induce the cell apoptosis.^121^

## Advance in the development of ADC

From the perspective of drug composition and technology characteristics, the development of ADC drugs could be usually subdivided into three generations (Table 3).

**Table 3:** The evolution of the ADC drug development

	First-generation ADC	Second-generation ADC	Third-generation ADC
Antibodies	Mouse-original or chimeric humanized antibodies	Humanized antibodies	Fully humanized antibodies or Fabs
Linkers	Unstable	Improved stability: cleavable and non-cleavable linkers;	Stable in circulation; precise control drugs release into tumor sites
Payloads	Low potency, including calicheamicin, duocarmycin and doxorubicin	Potency, such as auristatins and mytansinoids	High potency, such as PBDs, and tubulysin, and novel payloads like immunomodulators
Conjugation methods	Random lysines	Random lysines and reduced interchain cysteines	Site-specific conjugation
DAR	Uncontrollable (0–8)	4–8	2–4
Representative drugs	Gemtuzumab ozogamicin and inotuzumab ozogamicin	Brentuximab vedotin and ado-trastuzumab emtansine	Polatuzumab vedotin, enfortumab vedotin, and fam-trastuzumab deruxtecan
Advantages	• Specific targeting • Increase therapeutic window to some extent	• Improved targeting ability • More potent payloads • Lower immunogenicity	• Higher efficacy though in cancer cells with low antigen; • Improved DAR along with improved stability and PK/PD; • More potent payloads; • Less off-target toxicity
Disadvantages	• Heterogeneity; • Lack of efficacy; • Narrow therapeutic index; • Off-target toxicity as premature drug loss; • High immunogenicity	• Heterogeneity; • Fast clearance for high DARs; • Off-target toxicity as premature drug loss; • Drug resistance	• Possible toxicity due to highly potent payloads; • Catabolism may be different across species • Drug resistance

### The first-generation ADCs

In the early stage, ADC, such as BR96-doxorubincin, mainly consisted of a conventional chemotherapy drug conjugated to a mouse derived antibody through a non-cleavable linker.^31,122^ The potency of those ADCs was not superior to free cytotoxic drugs and the immunogenicity was frequently a concern.^123^ Later on, the use of much more potent cytotoxic agents in combination of humanized mAbs resulted in greatly improved efficacy and safety and thus market approval for the first-generation ADCs, including gemtuzumab ozogamicin and inotuzumab ozogamicin. In these two products, humanized mAbs of the IgG4 isotype were used and conjugated to the potent cytotoxic calicheamicin through the acid-labile linkers.^124,125^ The system is, however, not flawless. For example, acidic conditions can be appeared in other parts of the body and the linkers in first-generation ADC can also be found to hydrolyze slowly in the systemic circulation (pH 7.4, 37 °C), resulting in the uncontrollable release of toxic payload and unexpected off-target toxicity.^26^ Secondly, calicheamicin is hydrophobic that easy to cause antibody aggregation, accounting for the emergence of some defects, like short half‐life, faster clearance, and immunogenicity.^126,127^ Moreover, the conjugation of first-generation ADC is based on the stochastic conjugation via the lysine and cysteine residues, resulting in a group of highly heterogeneous mixtures with variable DARs.^128^ The DAR play a crucial role for the potency of an ADC.^129^ The inconsistent DAR exerts an influence on pharmacokinetic and pharmacodynamic (PK/PD) parameters and therapeutic index of ADC drugs.^130,131^ Consequently, the first-generation ADCs demonstrate suboptimal therapeutic windows and need further improvement.

### The second-generation ADCs

The second generation ADCs represented by brentuximab vedotin and ado-trastuzumab emtansine were subsequently launched after optimization of mAbs isotypes, cytotoxic payloads, as well as linkers. Both these two ADCs are based on the IgG1 isotype mAbs, which are more suitable for bioconjugation with small-molecule payloads and high cancer cell targeting ability compared to IgG4.^132,133^ Another major breakthrough in the second-generation ADC is the use of more effective cytotoxic drugs, such as auristatins and mytansinoids, with improved the water solubility and coupling efficiency.^53^ More payload molecules can thus be loaded onto each mAb without inducing antibody aggregation. In addition to the improvements with regards to the antibody carrier and cytotoxic payload, the linkers in the second generation ADCs are also improved to achieve better plasma stability and homogeneous DAR distribution.^134^ Overall, improvements in all these three elements result in better clinical efficacy and safety of the second generation ADCs. Nevertheless, there remain a number of unmet needs, such as insufficient therapeutic windows due to off-target toxicity, and aggregation or rapid clearance in those ADCs with high DAR. When DAR is over 6, the ADC demonstrates a high hydrophobicity and tends to decrease ADC potency due to faster distribution and clearance in vivo.^135–137^ In this context, the optimization of DAR by site-specific conjugation, along with continuous optimization of mAbs, linkers and payloads turn out to be the key for successful development of the third-generation ADCs.

### The third-generation ADCs

The third generation ADC are represented by polatuzumab vedotin, enfortumab vedotin, fam-trastuzumab deruxtecan and later approved ADCs. Benefitted from the introduction of site-specific conjugation technology, the homogenous ADCs with well-characterized DARs (2 or 4) and desired cytotoxicity were produced.^130^ ADCs with consistent DARs show less off-target toxicity and better pharmacokinetic efficiency.^138^ Moreover, fully humanized antibodies instead of chimeric antibodies are utilized in the third generation to reduce immunogenicity. In addition, antigen-binding fragments (Fabs) are being developed to replace intact mAbs in a number of ADC candidates since Fabs are more stable in systemic circulation and may be internalized more readily by cancer cells.^139^ Besides, more potent payloads such as PBD, tubulysin, and immunomodulator with novel mechanisms, have been developed to conjugate with antibodies.^26^ Although the linkers types in the third generation did not show any updates, some novel entities such as the Fleximer platform have been developed and used to conjugate varied payloads.^140,141^ In order to avoid the disturbance of immune system and improve retention time in blood circulation, more hydrophilic linker modulation such as PEGylation is employed in the third-generation of ADC.^142,143^ The hydrophilic linkers also provide utility in balancing highly hydrophobicity of certain cytotoxic payloads such as PBD, bearing in mind that ADCs with hydrophobic payloads are often prone to aggregation.^144^ Collectively, the third-generation ADC has lower toxicity and higher anticancer activity, as well as higher stability, allowing patients to receive better anticancer therapeutics.

## Clinical development of ADCs

### Approved ADC drugs

With several decades of efforts to optimize the key components, over 100 ADCs are currently under clinical development, and as of December 2021, a total of 14 ADC drugs have received the marketing approval in different countries worldwide. Coincidently half of the approved ADCs are mainly used against hematological malignancies and the rest are mainly prescribed for solid tumors. An overview of these ADCs including their molecular design, initial approval years, marketed company, approved countries and approved indications is shown in Table 4.

**Table 4:** Summary of antibody–drug conjugates approved for market worldwide for clinical use, as of December 2021.

Drugs (Company)	Trade Names	Target antigens	Linkers	Payloads	Average DAR	Approved Countries	Approved Date	Approved indications
Hematological malignancies								
Gemtuzumab ozogamicin (Pfizer)	Mylotarg^®^	CD33	hydrazone	N-acetyl-γ-calicheamicin	2–3	FDA/EMA/PMDA	2000/5/17; 2017/9/1	newly-diagnosed CD33-positive AML to include pediatric patients 1 month and older.
Brentuximab vedotin (Seagen)	Adcetris^®^	CD30	mc-VC-PABC	MMAE	4	FDA/EMA/PMDA/NMPA	2011/8/19	R/R CD30 positive HL and systemic ALCL; in combination with chemotherapy including the treatment of certain types of PTCL and previously untreated stage III or IV cHL.
Inotuzumab ozogamicin (Pfizer)	Besponsa^®^	CD22	hydrazone	N-acetyl-γ-calicheamicin	5–7	FDA/EMA/PMDA	2017/6/28	adults with R/R B-cell precursor ALL.
Moxetumomab pasudotox (AstraZeneca)	Lumoxiti^®^	CD22	mc-VC-PABC	PE38	NA	FDA/EMA	2018/9/13	adult patients with R/R HCL who have previously failed to receive at least two systemic therapies (including purine nucleoside analogs).
Polatuzumab vedotin (Roche)	Polivy^®^	CD79B	mc-VC-PABC	MMAE	3.5	FDA/EMA	2019/6/10	in combination with bendamustine plus rituximab for the treatment of patients with R/R DLBCL, who have received at least two prior therapies.
Belantamab mafodotin (GSK)	Blenrep^®^	BCMA	mc	MMAF	4	FDA/EMA	2020/8/5	adult patients with R/R MM who have received at least four treatments, including anti-CD38 monoclonal antibodies, proteasome inhibitors and immunomodulators
Loncastuximab tesirine (ADC Therapeutics)	Zynlonta^®^	CD19	dipeptide	PBD dimer (SG3199)	2.3	FDA	2021/4/23	adult patients with R/R large B-cell lymphoma after two or more lines of systemic therapy, including DLBCL not otherwise specified, DLBCL arising from low grade lymphoma and high-grade B-cell lymphoma
Solid Tumors								
Ado-trastuzumab emtansine (Roche)	Kadcyla^®^	HER2	SMCC	DM1	3.5	FDA/EMA/PMDA/NMPA	2013/2/22	adjuvant treatment of patients with HER2-positive early breast cancer who have residual invasive disease after neoadjuvant taxane and trastuzumab-based treatment.
Enfortumab vedotin (Seagen)	Padcev^®^	Nectin-4	mc-VC-PABC	MMAE	3.8	FDA	2019/12/18	locally advanced or metastatic urothelial cancer who have previously received platinum chemotherapy and a PD-L1/PD-1 inhibitor
Fam-trastuzumab deruxtecan (Daiichi Sankyo)	Enhertu^®^	HER2	tetrapeptide	DXd	7–8	FDA/EMA/PMDA	2019/12/20	adult patients with unresectable or metastatic HER2-positive breast cancer who have received two or more prior anti-HER2 based regimens in the metastatic setting; locally advanced or metastatic HER2-positive gastric or gastroesophageal junction adenocarcinoma who have received a prior trastuzumab-based regimen.
Sacituzumab govitecan (Immunomedics)	Trodelvy^®^	Trop-2	CL2A	SN38	7.6	FDA	2020/4/22	patients with unresectable locally advanced or metastatic TNBC who have received two or more prior systemic therapies, at least one of them for metastatic disease.
Cetuximab sarotalocan (Rakuten Medical)	Akalux^®^	EGFR	NA	IRDye700DX	1.3–3.8	PMDA	2020/9/25	unresectable locally advanced or recurrent HNSCC
Disitamab vedotin (RemeGen)	Aidixi^®^	HER2	mc-VC-PABC	MMAE	4	NMPA	2021/6/8	patients with locally advanced or metastatic gastric cancer (including gastroesophageal junction adenocarcinoma) who have received at least 2 types of systemic chemotherapy
Tisotumab vedotin (Genmab/Seagen)	Tivdak^®^	TF	mc-VC-PABC	MMAE	4	FDA	2021/9/20	adult patients with recurrent or metastatic cervical cancer with disease progression on or after chemotherapy, which is the first and only approved TF-directed ADC therapy

*FDA* US Food and Drug Administration, *EMA* European Medicines Agency, *PMDA* Pharmaceuticals and Medical Devices Agency of Japan, *NMPA* National Medical Products Administration of China, *DAR* Drug-to-Antibody ratio, *R/R* relapsed or refractory, *AML* acute myeloid leukemia, *mc-VC-PABC* maleimidocaproyl-valine-citrulline-p-aminobenzoyloxycarbonyl, *MMAE* monomethyl auristatin E, *MMAF* monomethyl auristatin-F, *HL* Hodgkin lymphoma, *ALCL* anaplastic large cell lymphoma, *SMCC* succinimidyl‐4‐(N‐maleimidomethyl)cyclohexane‐1‐carboxylate, *DM1* derivative of maytansine 1, *HER2* human epidermal growth factor receptor 2, *cHL* classical Hodgkin lymphoma, *PTCL* peripheral T-cell lymphomas, *MM* multiple myeloma, *PE38* a 38kD fragment of Pseudomonas exotoxin A, *DLBCL* diffuse large B-cell lymphoma, *PD-L1* programmed cell death-ligand 1, *PD-1* programmed cell death protein-1, *DXd* Exatecan derivative for ADC, *CL2A* a cleavable complicated PEG8- and triazole-containing PABC-peptide-mc linker, *SN38* active metabolite of irinotecan, *HCL* hairy cell leukemia, *TNBC* triple-negative breast cancer, *HNSCC* head and neck squamous cell carcinoma, *BCMA* B-cell maturation antigen, *EGFR* epidermal growth factor receptor, *GSK* GlaxoSmithKline Inc., *PBD* pyrrolobenzodiazepine, *TF* tissue factor, *mc* maleimidocaproyl.

#### Hematological malignancies

##### Gemtuzumab ozogamicin (Mylotarg^®^, Pfizer)

Gemtuzumab ozogamicin is the first ADC type of therapeutics approved for clinical use in the world. It consists of an engineered humanized monoclonal IgG4 antibody that targets CD33 and a cytotoxic N‐acetyl-γ-calicheamicin via a cleavable hydrazone linker. Gemtuzumab ozogamicin has an average DAR of 2–3. With a response rate of 26%, it was firstly approved by the FDA for use in patients with relapsed or refractory (r/r) CD33 positive AML in first relapse who were over 60 years and were not suitable for other conventional chemotherapies.^145^ Roughly 85–90% of adult and pediatric AML are CD33 positive.^146^ After binding with CD33 antigens and internalization by cancer cells, followed a hydrolysis of hydrazone bond to release calicheamicin. And the calicheamicin could also diffuse to the other cancer cells nearby, which induces bystander killing effect to those antigen-negative cancer cells.

However, the hydrazone based linker in gemtuzumab ozogamicin is not perfectly stable, resulting in the premature release of calicheamicin in the plasma and increase off-target toxicity.^147^ The results from Study SWOG S0106A have showed that a higher rate of severe fatal toxicity was observed but without significant clinical benefit response in the patients with combination therapy (gemtuzumab ozogamicin with standard daunorubicin and cytarabine chemotherapy) compared with those receiving chemotherapy (daunorubicin and cytarabine) alone.^148^ Hence, Pfizer Inc. voluntarily withdrew this product from market in October 2010.

Afterwards, the efficacy and safety of gemtuzumab ozogamicin were re-evaluated using a lower recommended dosage (3 mg/m^2^) than what was approved in 2000 (9 mg/m^2^). The gemtuzumab ozogamicin combined with chemotherapy were investigated in the clinical trial ALFA-0701, a multicenter, randomized, open-label phase 3 study.^149^ A total of 271 patients (50–70 years old) with newly-diagnosed AML were randomly assigned to receive induction therapy consisting of daunorubicin (60 mg/m^2^) and cytarabine (200 mg/m^2^) with (*n* = 135) or without (*n* = 136) gemtuzumab ozogamicin. The event free survival (EFS) was used as primary endpoint and patients receiving gemtuzumab ozogamicin combined with chemotherapy showed a longer EFS than those receiving chemotherapy only, and the median EFS were 17.3 months and 9.5 months, respectively (HR = 0.56 [95% CI: 0.42–0.76]). The Grade ≥ 3 adverse events (AEs) occurred in two groups (gemtuzumab ozogamicin combined with chemotherapy *vs* chemotherapy only) included infection (47% *vs* 39%), hemorrhage (18% *vs* 9%), and veno-occlusive liver disease (2% *vs* 0%).

In addition, the safety and efficacy evaluation of gemtuzumab ozogamicin as monotherapy was performed in AML-19 and MyloFrance-1 studies.^20,150^ In AML-19, the overall survival (OS) was used as for assessment of efficacy. As a result, the median OS was 4.9 months *v.s*. 3.6 month (gemtuzumab ozogamicin *v.s*. best supportive care, HR = 0.69 [95% CI: 0.53–0.90]). The Grade ≥ 3 AEs occurred in over 5% patients were infection (35%), febrile neutropenia (18%), bleeding (13%), fatigue (12%), liver (7%), and cardiac (6%). And in MyloFrance-1, 26% complete remission (CR) rate was observed. The grade ≥ 3 AEs occurred in over 5% patients included sepsis (32%), fever (16%), rash (11%), pneumonia (7%), bleeding (7%). Based on the overall positive outcomes achieved in above three investigator-led clinical trials, thus the *Mylotarg*^*®*^ was re-approved by the FDA in 2017.^149,151,152^ Recently, a new indication of gemtuzumab ozogamicin was approved by the FDA for the treatment of newly-diagnosed CD33-positive AML to include pediatric patients 1 month and older.^153^ The rare listing experience gained from the withdrawal and re-approval of gemtuzumab ozogamicin provides important reference for the development and clinical trials design for ADCs.

##### Brentuximab vedotin (Adcetris^®^, Seagen)

Brentuximab vedotin also known as SGN-35, is the second ADC drug received market approval by the FDA in 2011 for the treatment of r/r CD30 positive Hodgkin lymphoma (HL) and systemic anaplastic large cell lymphoma (sALCL). It is composed of a chimeric IgG1 monoclonal antibody brentuximab that targets CD30, a maleimide attachment group, a cleavable dipeptide linker (maleimidocaproyl-valine-citrulline-p-aminobenzoyloxycarbonyl or mc-VC-PABC), and antimitotic agent MMAE.^132^ The average DAR of brentuximab vedotin was 4. Through selective targeting to CD30 antigen, a hallmark of HL and ALCL, brentuximab vedotin is internalized via a clathrin‐dependent mechanism and transferred into endosomes and lysosomes where the linker is hydrolyzed by cysteine proteases, like cathepsin B. The released free MMAE then targets to tubulin to inhibit its polymerization, causing cell cycle arrest and cell apoptosis.^132^ In virtue of bystander effects, brentuximab vedotin is able to take effect for those antigen-negative cancer cells.

Compared to the hydrazine linker in gemtuzumab ozogamicin, the dipeptide-based linker in brentuximab vedotin shows better stability under physiologic conditions, thus premature release of the cytotoxic payload in the plasma is minimal. Moreover, the linker is sensitive to cysteine proteases that can facilitate efficient release of the payload inside cancer cells to ensure the killing effects.^154^ Another improvement of brentuximab vedotin is the use of the more potent cytotoxic payload, MMAE. It is the synthetic derivative of natural product Dolastatin 10 and functions as a ultrapotent antimitotic agent that induces cell cycle arrest by blocking tubulin polymerization.^155^ It is widely used as the payload in several ADCs, such as polatuzumab vedotin, enfortumab vedotin, and disitamab vedotin.^156^

The effectiveness of brentuximab vedotin for HL and sALCL was investigated in two single-arm phase-II trials with 73% and 86% of the patients achieved objective response, respectively, thus the FDA granted the accelerated approval of *Adcetris*^*®*^ for r/r HL and sALCL in 2013.^157,158^ The effectiveness of brentuximab vedotin in patients with HL was evaluated in a pivotal, phase II, single-arm, multicenter study involving 102 patients with r/r HL after autologous stem cell transplant (SCT).^159^ The objective response rate (ORR) was used as primary endpoint. As a result, either a complete response (CR) or partial response (PR) was observed in 73% patients who received brentuximab vedotin (1.8 mg/kg) and the average response time of patients to treatment was 6.7 months. The AEs ≥ grade 3 occurring in ≥ 5% of patients were neutropenia (20%), peripheral sensory neuropathy (8%), thrombocytopenia (8%), and anemia (6%).^160^ While for sALCL, it was evaluated in a phase II, single-arm, multicenter study in 58 patients with r/r sALCL.^161^ Of the patients receiving brentuximab vedotin (1.8 mg/kg), 86% experienced either a complete or partial response and responded on average for 12.6 months. The severe AEs observed in patients with sALCL were similar with those with SCT.^162^

In November 2017, brentuximab vedotin received additional approval as a treatment for primary cutaneous anaplastic large cell lymphoma (pcALCL) or CD30-expressing mycosis fungoides (MF) who have received prior systemic therapy based on the positive data from a phase 3 study (ALCANZA), in which brentuximab vedotin demonstrated ORR lasting no less than four 4 months.^163^ Moreover, in 2018, two more clinical indications of brentuximab vedotin were approved in combination with chemotherapy including the treatment of certain types of peripheral T-cell lymphoma (PTCL) and previously untreated stage III or IV classical Hodgkin lymphoma (cHL).^164,165^

##### Inotuzumab ozogamicin (Besponsa^®^, Pfizer)

Inotuzumab ozogamicin, also known as CMC-544, consists of a humanized mAb targeting CD22 linked to a cytotoxic N‐acetyl-γ-calicheamicin with an average DAR of 5–7. CD22 is a cell surface antigen found in the majority (60–90%) of B-cell acute lymphoblastic leukemia (B-ALL).^166,167^ And binding to CD22 activates a series of downstream processes of the ADC, including internalization, linker hydrolysis and payload release, in a similar manner as seen in gemtuzumab ozogamicin. Through an open-label, randomized, international, multicenter phase 3 study (INO-VATE 1022), the safety and efficacy of inotuzumab ozogamicin was evaluated compared with investigator’s choice of chemotherapy in 326 adult patients with r/r B-ALL who had received one or two prior treatments.^168^ All the enrolled patients were randomly assigned to inotuzumab ozogamicin treatment or an alternative chemotherapy regimens including FLAG (fludarabine, cytarabine and G-CSF), HIDAC (high dose cytarabine), mixture of cytarabine and mitoxantrone. The percentage of patients with no evidence of disease and full recovery of blood counts after treatment was used as primary indicator in this study. The results demonstrated that 35.8% patients with inotuzumab ozogamicin treatment achieved CR while 17.4% was observed in alternative chemotherapy group.^168,169^ The AEs ≥ grade 3 in the inotuzumab ozogamicin arm include neutropenia (47%), thrombocytopenia (41%), leukopenia (27%), and febrile neutropenia (27%).^168^ Based on these positive results, in August 2017, the FDA approved *Besponsa*^*®*^ for marketing, the first and so far the only CD22-directed ADC for the treatment of adults with r/r B-cell precursor ALL.

##### Moxetumomab pasudotox (Lumoxiti^®^, AstraZeneca)

Hairy cell leukemia (HCL) is a rare hematological malignancy, which is characterized by splenomegaly, hemorrhage, and an accumulation of abnormal B lymphocytes.^170^ In addition to B-ALL, CD22 also expressed in B cells in HCL and is thus used as a target for treatment. Instead of using small-molecule payload, moxetumomab pasudotox consists of moxetumomab targeting CD22 conjugated to a 38kD fragment of *Pseudomonas* exotoxin A (PE38).^171^ CD22 is expressed on mature B cells and much more intensively on 100 % of hairy cells, which provides an ideal therapeutic target for the treatment of HCL.^172,173^ Upon binding to CD22, moxetumomab pasudotox is internalized, cleaved and released catalytic domain of the exotoxin inside cancer cells, which inhibits the translation of proteins leading to apoptosis.

A phase 3 clinical study (Study 1053) for moxetumomab pasudotox enrolled 80 patients with histologically confirmed HCL or HCL variant requiring treatment based on presence of cytopenias or splenomegaly and who had received prior treatment with at least two systemic therapies (including one purine nucleoside analog).^174^ The patients received moxetumomab pasudotox treatment (0.04 mg/kg) until the observation of CR, disease progression, or unacceptable toxicity. In the study, the ORR and the CR rate of moxetumomab pasudotox monotherapy was 75% (95% CI, 64–84) and 41% (95% CI, 30–53), respectively. Additionally, the durable complete response rate was 30% (95% CI, 20–41).^175^ The most commonly occurring grade 3–4 events were decreased lymphocyte (20%), anemia (10%), and asymptomatic hypophosphatemia (10%)..^175,176^ In September 2018, the FDA approved *Lumoxiti*^*®*^ of AstraZeneca for the treatment of adult patients with r/r HCL who have previously failed to receive at least two systemic therapies (including purine nucleoside analogs).^177^ This made moxetumomab pasudotox the first new drug approved for the treatment of HCL in the past 20 years, a remarkable milestone in the clinical treatment of HCL.

##### Polatuzumab vedotin (Polivy^®^, Roche)

Polatuzumab vedotin, also known as DCDS4501, contains a humanized antibody targeting CD79b linked to microtubule-disrupting MMAE via a protease-cleavable dipeptide linker (mc-VC-PABC) with an average DAR of 3.5.^178^ CD79b, a component of the B-cell receptor (BCR), is expressed on over 90% of B-cell non-Hodgkin lymphomas (nHL) malignancies and has been shown as a promising antibody target.^179,180^ Similar with brentuximab vedotin, upon administration, polatuzumab vedotin selectively binds to CD79b followed endocytosis and proteolytic cleavage to release MMAE that induces cell cycle arrest and cell death. In July 2019, *Polivy*^*®*^ was approved by the FDA to be used in combination with bendamustine plus rituximab for the treatment of r/r diffuse large B-cell lymphoma (DLBCL) in patients who have received at least two prior therapies.^181^ It was the first ADC for treatment of DLBCL which was the most common type of nHL.

The approval was based on the positive results from a global, randomized phase Ib/II GO29365 study that included 80 patients with r/r DLBCL after at least one prior regimen.^182^ The enrolled patients were randomly assigned to either polatuzumab vedotin (Pola, 1.8 mg/kg, intravenous infusion) in combination with bendamustine (B, 90 mg/m^2^ intravenously) and a rituximab (R, 375 mg/m^2^ intravenously) or BR alone for six 21-day cycles. CR rate and response duration were determined as study endpoints. As a result, 40% patients with Pola+BR were observed CR while 18% with BR treatment alone. Among patients with PR or CR to Pola+BR treatment, the percentages with response durations of over 6 months and 12 months were 64% and 48%, respectively. The grade 3–4 AEs in Pola+BR group include neutropenia (46%), thrombocytopenia (41%), amenia (28%), lymphopenia (12.8%), and febrile neutropenia (10.3%).^182^

##### Belantamab mafodotin (Blenrep^®^, GSK)

Belantamab mafodotin also known as GSK2857916, is a novel ADC composed of a humanized FC modified anti-BCMA mAb coupled with cytotoxic agent MMAF through a non-cleavable maleimidocaproyl (mc) linker. Belantamab mafodotin has an average DAR of 4. BCMA is a transmembrane glycoprotein specifically overexpressed on the surface of multiple myeloma (MM) cells.^183^ After binding to BCMA, belantamab mafodotin is rapidly internalized, degraded in lysosomes to release impermeable MMAF inside MM cells. MMAF, similar with MMAE, is also a mitotic inhibitor. It could inhibit cell division by blocking microtubule polymerization, resulting in cell cycle arrest and inducing caspase-3-dependent apoptosis. Altogether, belantamab mafodotin effectively cause cell death in cancer cells overexpressed BCMA. In August 2020, the FDA approved *Blenrep*^*®*^ for the treatment of r/r MM. It is the first BCMA-targeted therapy for MM that was approved based on the results of the DREAMM-2 clinical trial, a two-arm, open-label, multicenter phase II study.^184^

In this study, a total of 221patients (aged ≥18 years) with r/r MM with disease progression after three or more lines of therapy and who were refractory to immunomodulatory drugs and proteasome inhibitors, and refractory or intolerant (or both) to an anti-CD38 monoclonal antibody with an Eastern Cooperative Oncology Group performance status of 0–2 were enrolled and randomly assigned (1:1) to received two different doses of belantamab mafodotin (2.5 mg/kg and 3.4 mg/kg, respectively) until disease progression or unacceptable toxicity. Efficacy was based on ORR and response duration. As a result, the data demonstrated that the treatment with belantamab mafodotin alone, the ORRs in 2.5 mg/kg arm and 3.4 mg/kg arm were 32% and 35%, respectively. A promising partial response (VGPR) was observed in 58% and 66% in the 2.5- and 3.4-mg/kg cohorts, respectively. The most common grade 3–4 AEs were keratopathy (27% in the 2.5 mg/kg cohort and 21% in the 3.4 mg/kg cohort), thrombocytopenia (20% and 33%), and anemia (20% and 25%).^184^

##### Loncastuximab tesirine (Zynlonta^®^, ADC Therapeutics)

Loncastuximab tesirine also known as ADCT-402, is consists of a humanized mAb targeting CD19 conjugated to PBD dimer via a cleavable (valine-alanine dipeptide) maleimide type linker.^185^ The average DAR of loncastuximab tesirine was approximately 2.3.^186^ PBD dimer is a new generation cytotoxic payload for the ADC development.^187^ It irreversibly binds to DNA and cause strong inter strand cross-linking that prevents DNA strand separation, thus destroying necessary DNA metabolic processes and finally leading to cell death.^188^ It does not depend on the cell division cycle and the damage is not easy to restore, showing better cytotoxicity.^189^ In April 2021, *Zynlonta*^*®*^ received accelerated approval by the FDA for the treatment of adult patients with r/r large B-cell lymphoma after two or more lines of systemic therapy, including diffuse large B-cell lymphoma (DLBCL) not otherwise specified (NOS), DLBCL arising from low grade lymphoma and high-grade B-cell lymphoma. Loncastuximab tesirine is the first and so far the only CD19 targeted ADC that approved for patients with r/r DLBCL as a single agent.

The approval of *Zynlonta*^*®*^ was based on the data from LOTIS-2 study, a multicenter, open-label, single-arm, phase 2 trial.^190^ A total of 145 adult patients with r/r DLBCL or high-grade B-cell lymphoma after at least two prior systemic regimens were enrolled and treated with loncastuximab tesirine (0.15 mg/kg). The overall ORR was used to access the main efficacy of loncastuximab tesirine. It was shown that for the patients received with loncastuximab tesirine, the ORR reached 48.3% (95% CI: 39.9–56.7) with 24.1% (95% CI: 17.4–31.9) of CR. After a median follow-up of 7.3 months, median response duration was 10.3 months (95% CI: 6.9, NE). Of the 70 patients who achieved objective responses, 36% were censored for response duration prior to 3 months. The most common grade≥3 AEs were neutropenia (26%), thrombocytopenia (18%), and increased gamma-glutamyltransferase (17%).^190^

#### Solid tumors

##### Ado-trastuzumab emtansine (Kadcyla^®^, Roche)

About 15%-20% of breast cancer patients show human epidermal growth factor receptor 2 (HER2) positive overexpression with a higher invasiveness.^191,192^ Ado-trastuzumab emtansine, also known as T-DM1, is an ADC drug targeting HER2 and the first ADC to be approved in a solid tumor. It is consisted of a humanized mAb targeting HER2 linked to DM1 through a non-cleavable linker (succinimidyl‐4‐(N‐maleimidomethyl)cyclohexane‐1‐carboxylate, SMCC) with an average DAR of 3.5.^133^ The linker could keep the conjugate more stable in plasma circulation but release payloads after endocytosis in the HER2‐positive cancer cells. The complete digestion of trastuzumab by proteases in the lysosome allows the release of a DM1 containing metabolite, lysine-MCC-DM1, which shows similar cytotoxicity compared to free DM1. Furthermore, the lysine-MCC-DM1 is charged under physiological pH that it not applicable to exert the bystander effect. Therefore, T-DM1 targets and causes the death of the antigen positive cancer cells only. In addition, T-DM1 was shown a similar mechanisms with trastuzumab that it could inhibit HER2 signaling pathway, induce ADCC and CDC effects.^193^

In 2013, *Kadcyla*^*®*^ obtained the market approval by the FDA for use as a single drug in the treatment of HER2 positive metastatic breast cancer patients who had previously received *Herceptin*^*®*^ (trastuzumab) and taxane chemotherapy. The approval was based on the positive outcomes from the phase 3 study (EMILIA).^194,195^ A total of 991 adult patients with HER2-positive unresectable, locally advanced or metastatic breast cancer previously treated with trastuzumab and a taxane were enrolled and randomized to receive T-DM1 (3.6 mg/kg) or lapatinib plus capecitabine. The PFS and OS were used as primary endpoints. In the final descriptive analysis, the median PFS of T-DM1 arm was 9.6 months while 6.4 months was determined in lapatinib plus capecitabine arm (*p* < 0001). And median OS of T-DM1 arm and lapatinib plus capecitabine arm were 30.9 months and 25.1 months, respectively. The most common grade ≥3 AEs in T-DM1 arm were: thrombocytopenia (12.9%), increased AST (4.3%), and increased ALT (2.9%).^194^ It is worth noting that there is warning label for cardiotoxicity to T-DM1 due to the observation of left ventricular ejection fraction (LVEF) decrease.^196^

Moreover, based on the positive results from the phase 3 study (KATHERINE), the FDA extended the approval to *Kadcyla*^*®*^ for adjuvant treatment of patients with HER2-positive early breast cancer (EBC) who have residual invasive disease after neoadjuvant taxane and trastuzumab-based treatment in May 2019.^197,198^ A total of 1486 patients met criteria were enrolled in the study and randomly assigned to treat with T-DM1 or trastuzumab. As the primary endpoint of this study, invasive disease-free survival (IDFS) was improved significantly in group who received T-DM1 compared to treated with trastuzumab by 50%. At three years, 88.3% of patients treated with T-DM1 did not relapse compared to 77.0% treated with trastuzumab.^198^

##### Enfortumab vedotin (Padcev^®^, Seagen)

Enfortumab vedotin also known as ASG-22ME, is approved by the FDA for the treatment of adult patients with locally advanced or metastatic urothelial cancer.^199^ It is composed of a fully human anti-nectin-4 IgG1 kappa monoclonal antibody (AGS-22C3), linked to MMAE via a protease-cleavable linker (MC-VC-PABC) and has an average DAR of approximately 3.8.^200^ Nectin-4 is a transmembrane protein belonging to the nectin family, which plays a crucial role for cell proliferation, migration and adhesion.^201,202^ It has been found to be abundantly expressed in in several malignancies, especially in urothelial carcinoma. Through immunohistochemical analysis, 60% of bladder tumor specimens were observed a strong staining while a limited staining showed in normal tissue.^200^ As such, it has emerged as a compelling target for novel molecular design of ADCs. Enfortumab vedotin is the first and so far the only FDA-approved ADC that targeted nectin-4. The accelerated approval was firstly granted by the FDA in December 2019 while a regular approval was further granted in September 2021 based on results from an open-label, randomized, multicenter phase 3 study (EV-301).^203–205^

In EV-301 study, a total of 608 patients with locally advanced or metastatic urothelial cancer who received a prior PD-1 or PD-L1 inhibitor and platinum-based chemotherapy were enrolled and randomized equally to treat with either enfortumab vedotin (1.25 mg/kg) or alternative chemotherapy (docetaxel, paclitaxel, or vinflunine). The OS and PFS were respectively used as primary endpoint and secondary endpoints for evaluation of efficacy. Compared with alternative chemotherapy, enfortumab vedotin achieved a remarkable efficacy with a significantly prolonged median OS (12.9 *v.s*. 9.0 months) and median PFS (5.6 *v.s*. 3.7 months).^199,204^ Grade ≥3 AEs that occurred in at least 5% of patients were maculopapular rash (7.4%), fatigue (6.4%), and decreased neutrophil count (6.1%) in the enfortumab vedotin group.^204^

##### Fam-trastuzumab deruxtecan (Enhertu^®^, Daiichi Sankyo)

Enhertu, also known as DS-8201 or T-DXd, is HER2-targeted ADC for the treatment of adult patients with unresectable or metastatic HER2-positive breast cancer who have received two or more prior anti-HER2 based regimens in the metastatic setting.^206^ It is composed of a humanized HER2 antibody (trastuzumab) conjugated to a novel topoisomerase I inhibitor (DXd) as payload through a enzymatically cleavable tetrapeptide-based linker with an average DAR of 7–8. DXd was reported to be more potent than SN-38, the active form of the irinotecan and the higher potency of DXd ensures the efficacy when it was used as payload in ADCs.^74^ Another improvement of DS-8201 is the utilization of novel tetrapeptide-based linker technology, which could keep more stable in plasma to reduce the risk of systemic toxicity.^207^

In December 2019, *Enhertu*^*®*^ was approved by the FDA based on the positive results from a single-arm, multicenter, phase 2 DESTINY-Breast01 study.^208^ 184 female patients with HER2-positive, unresectable and/or metastatic breast cancer (mBC) who had received two or more prior anti-HER2 therapies were enrolled in the study. The primary endpoint was ORR and response duration. As a result, ORR of patients received DS-8201 (5.4 mg/kg) was 60.3% (95% CI: 52.9, 67.4), with a 4.3% CR rate and a 56% PR rate. The median response duration was 14.8 months, and the median duration of PFS was 16.4 months.^207^ The most common AEs of grade ≥ 3 that occurred in more than 5% of the patients were a decreased neutrophil count (20.7%), anemia (8.7%), nausea (7.6%), a decreased white-cell count (6.5%), a decreased lymphocyte count (6.5%), and fatigue (in 6.0%).^207^

Moreover, The recently updated data of DESTINY-Breast03, a global, head-to-head, randomized, open-label, pivotal phase 3 trial, demonstrated that DS-8201 had an significant superiority over T-DM1.^209,210^ In detail, 524 patients with HER2 + mBC previously treated with trastuzumab and taxane were enrolled and randomized (1:1). The primary endpoint was PFS and secondary endpoints including OS, ORR, and duration of response were used. As a result, the median PFS was not observed for DS-8201 *v.s*. 6.8 month for T-DM1. And the median response duration was 14.3 month for DS-8201 compared to 6.9 month treated with T-DM1. In addition, another head-to-head study (DESTINY-Breast09) was carrying out for the comparison between DS-8201 and trastuzumab.^211^

The efficacy and safety of DS-8201 in patients with metastatic NSCLC with HER2 mutations were also assessed in DESTINY-Lung01 study.^212^ Among 91 enrolled patients, 55% had a confirmed OR at a median follow-up duration of 13.1 months. The median PFS duration was 8.2 months, with a median OS duration of 17.8 months. The adverse events (grade ≥3) were observed in 46% of patients, including neutropenia (in 19%) and anemia (in 10%). The clinical observations have also raised a concern regarding potential lung toxicity of DS-8201. It is noteworthy that the interstitial lung disease (ILD) was observed in 26% (23 in 91) of patients and two patients died of treatment-related ILD. ILD is a group of respiratory diseases affecting the interstitium of the lungs, which would disrupt the repair damage process of our body and block oxygen from participating in blood circulation.^213^ Hence, more careful attention to ILD and appropriate training of clinicians for the identification and management of this toxic effect are required in the follow-up clinical trials.

Since the launch of DS-8201, its clinical potential is still expanding and deepening. The new indication of DS-8201 for gastric cancer has also been approved.^214^ And the line of treatment of breast cancer is moving forward, which is constantly providing better treatment options for patients.

##### Sacituzumab govitecan (Trodelvy^®^, Immnomedics)

Sacituzumab govitecan, also known as IMMU-132, is an ADC composed of a humanized monoclonal antibody targeting Trop-2 conjugated to a topoisomerase I inhibitor (SN-38) using a hydrolysable linker (CL2A) and has an average DAR of approximately 7.6. Trop-2, is a 40-kDa glycoprotein that plays a role as transducer of intracellular calcium signaling.^215,216^ An overexpression of Trop-2 was observed in the majority of solid tumors, including triple-negative breast cancer (TNBC).^217^ Theoretically, the overexpression of Trop-2 protein is related to the strong invasiveness and poor outcomes, which makes Trop-2 as an ideal broad-spectrum therapeutic target.^218^ In sacituzumab govitecan, SN-38 is the active form of irinotecan that causes frequent DNA single strand breaks by inhibiting DNA topoisomerase I and eventually leads to cell death.^73^ In term of the CL2A, it links SN-38 and Trop-2 antibody and is the most breakthrough design of sacituzumab govitecan as the third generation ADC. This well-designed connector improves the binding ratio of Trop-2 antibody to SN-38, with higher toxic concentration in tumor but lower concentration in non-target with shorter half-life.^219^ Through optimization of the stability of linker, it can not only release SN-38 in the target tumor cells, but also achieve the bystander effects to kill neighboring cancer cells that are difficult to target.^220^

In April 2020, sacituzumab govitecan received accelerated approval by the FDA for the treatment of patients with unresectable locally advanced or metastatic TNBC who have received two or more prior systemic therapies, at least one of them for metastatic disease. It is the first anti-Trop-2 ADC approved by the FDA for metastatic TNBC. And the clinical benefit of sacituzumab govitecan was further confirmed in a following multicenter, open-label, randomized trial (ASCENT), which promotes the regular approval by the FDA. The ASCENT study was performed in 529 patients with unresectable locally advanced or mTNBC who had relapsed after at least two prior chemotherapies, one of which could be in the neoadjuvant or adjuvant setting, if progression occurred within 12 months.^221^ The enrolled patients were randomized into two groups, receiving sacituzumab govitecan (*n* = 267, 10 mg/kg) and single agent chemotherapy (*n* = 262, capecitabine, eribulin, vinorelbine, or gemcitabine), respectively. The primary efficacy endpoint was PFS in in patients without brain metastases. As a result, median PFS for patients receiving sacituzumab govitecan was 4.8 months *v.s*. 1.7 months in those receiving chemotherapy. And the median OS was 11.8 months (sacituzumab govitecan) and 6.9 months (chemotherapy), respectively.^222^ The incidences of severe AEs of sacituzumab govitecan with grade ≥ 3 included neutropenia (51%), leukopenia (10%), diarrhea (10%), anemia (8%), and febrile neutropenia (6%).^222,223^

##### Cetuximab sarotalocan (Akalux^®^, Rakuten Medical)

Cetuximab sarotalocan also known as RM-1929, is a novel ADC consisting of an anti-EGFR chimeric monoclonal antibody, cetuximab, conjugated with IRDye^®^700DX, a near-infrared photosensitizing dye.^224^ The average DAR of cetuximab sarotalocan was in the 1.3–3.8 range. EGFR is abundantly expressed on the surface of multiple kinds of solid tumors, including head and neck squamous cell carcinomas (HNSCC), esophageal cancer, lung cancer, colon cancer, pancreatic cancer and other solid tumors.^225^ Cetuximab sarotalocan could target EGFR and be activated locally by the red laser released by the optical fiber after targeted combination with cancer cells, resulting in the cell death. It not only uses antibody mediated targeted delivery to achieve high tumor specificity, but also employs laser activated biophysical mechanism to accurately induce the rapid death of cancer cells without damaging surrounding normal tissues.^226^

In September 2019, cetuximab sarotalocan was approved by the Pharmaceuticals and Medical Devices Agency (PMDA) of Japan for the treatment of unresectable locally advanced or recurrent HNSCC. The approval was supported by the positive data from a multicenter, open-label phase 2a trial.^227^ A total of 30 patients with locoregional, HNSCC who could not be satisfactorily treated with surgery, radiation, or platinum chemotherapy were enrolled in the study. After administration of RM1929 for 24 h, the non-thermal red light was used to illuminate the tumor areas. The results demonstrated that the treatment with cetuximab sarotalocan, the ORR was 28% including 14% CR, and the median PFS and median OS were 5.7 months and 9.1 months, respectively.^227,228^ The most common AEs of grade ≥3 included skin reaction (18%), paronychial cracking (12%), and allergic reaction (3.5%).^227,228^ To date, cetuximab sarotalocan has not been approved outside of Japan and is currently running a global Phase 3 trial.

##### Disitamab vedotin (Aidixi^®^, RemeGen)

Disitamab vedotin also known as RC48, is the third listed HER2-targeted ADC, which consists of a novel humanized HER2 antibody, a cathepsin cleavable linker (mc-VC-PABC), and a cytotoxic agent, MMAE.^229^ The average DAR of RC48 is approximately 4. The antibody used in disitamab vedotin has a higher affinity to HER2, showing a more potent antitumor activity in animal models.^230^ On June 15, 2021, disitamab vedotin was conditionally approved by the National Medical Products Administration (NMPA) of China for the treatment of patients with locally advanced or metastatic gastric cancer (including gastroesophageal junction adenocarcinoma) who have received at least 2 types of systemic chemotherapy. It is the first ADC drug developed by China that approved for marketing. The approval was supported by the results of RC48-C008 study, which demonstrates a clinically meaningful response and survival benefit for patients received RC48.^231^

RC48-C008 study is a single-arm, multicenter, open label, phase 2 clinical trial with enrollment of 127 patients with histologically confirmed gastric or gastro-esophageal junction cancer, HER2-overexpression post-to ≥2 prior systemic treatment. In the trial, participating patients receive disitamab vedotin (2.5 mg/kg) until investigator-assessed loss of clinical benefit or unacceptable toxicity. ORR was used as primary outcome along with PFS and OS as secondary outcomes. The data showed that the ORR of overall patients was 18.1% (95% CI: 11.8–25.9%). And sub-group ORR was 19.4% and 16.9% for the participating patients post to 2 lines and ≥ 3 lines, respectively. Overall, the ORR of 111 patients who had received no less than 2 cycles of treatment was 20.7%. For all 127 patients, the median PFS was 3.8 months and the mOS was 7.6 months.^231^ Grade 3 or higher AEs were observed in 40 patients (32.0%), of which the most common were decreased neutrophil count (14.4%), decreased WBC count (14.4%), and anemia (5.6%).^232^

In addition, disitamab vedotin was also received conditional approval by NMPA in December 2021 for the second-line treatment of patients with HER2 positive locally advanced or metastatic urothelial cancer who have also previously received platinum-containing chemotherapy treatment. It is supported by the results of RC48-C005 Study, which was an open-label, multicenter, single-arm, non-randomized phase 2 study.^233^ 64 patients with HER2 overexpressing and locally advanced or metastatic urothelial cancer post to the failure of platinum, gemcitabine and taxane were enrolled and received RC48 (2 mg/kg) in the study. ORR was used as primary endpoint. As of Nov 30, 2020, the ORR was 55.6% (5/9), 50.0% (21/20) and 30.8% (4/13), in patients who had received 1 line, 2 lines and ≥ 3 lines treatment, respectively.

##### Tisotumab vedotin (Tivdak^®^, Genmab/Seagen)

Tisotumab vedotin is the most recently approved ADC drug with an average DAR of 4, which contains a fully humanized mAb binding to tissue factor (TF), a cleavable mc-VC-PABC linker, and an antimitotic agent, MMAE.^234^ TF plays an important role in tumor growth, angiogenesis and metastasis and is specifically overexpressed on several solid tumors.^235^ Tisotumab vedotin aims to target TF antigen on cancer cells and deliver cytotoxic agent MMAE directly into cancer cells. In addition, the bystander effect, ADCC and ADCP are also shown to be involved in the mechanism of action of tisotumab vedotin.^234^ In September 2021, *Tivdak*^*®*^ was received the approval by the FDA for adult patients with recurrent or metastatic cervical cancer with disease progression on or after chemotherapy, which is the first and only approved TF-directed ADC therapy.

The approval was supported by the findings from innovaTV 204 study, a multicenter, open-label, single-arm, phase 2 trial.^236^ A total of 101 patients who met criteria (recurrent or metastatic cervical cancer who previously received no more than two prior systemic regimens, including at least one prior platinum-based chemotherapy regimen, in the recurrent or metastatic setting) were enrolled in the study to received tisotumab vedotin 2 mg/kg every 21 days until disease progression or unacceptable toxicity occurred. The confirmed ORR was used as the main efficacy outcomes. Results showed the ORR was 24% (95% CI: 15.9%-33.3%) with a median response duration of 8.3 months (95% CI: 4.2, not reached).^237^ Grade 3 or higher AEs were observed in 28% patients, including neutropenia (3%), fatigue (2%), ulcerative keratitis (2%), and peripheral neuropathies (2%).^236^

### Late-phase ADC candidates

In addition to above 14 approved ADC drugs, hundreds of ADCs with the updated technology and novel indications are currently in clinical trials. Three represented ADC candidates in the phase-3 were discussed as following in this review to provide a conceptual snapshot.

#### Mirvetuximab soravtansine (ImmunoGen)

Mirvetuximab soravtansine (IMGN853) is composed of a humanized mAb targeting folate receptor alpha (FRα) conjugated to a potent cytotoxic DM4 by a cleavable linker (sulfo-SPDB). It has been granted by the FDA for the treatment of ovarian cancer as orphan drug designation.^238^ Through the innovative introduction two methyl groups at α site of disulfide bonds along with a sulfonyl group, the hydrophilicity of the linker is improved, which overcomes the disadvantage that the drugs prematurely release in the plasma circulation. In December 2015, an open-label, randomized Phase 3 trial (NCT02631876) were conducted to investigate the safety and efficacy of IMGN853 along with the selected single-agent chemotherapy in the treatment of women with platinum-resistant FRα positive advanced epithelial ovarian cancer, primary peritoneal cancer and/or fallopian tube cancer.^239^ It is the first FRα-targeting ADC candidate to enter into human clinical trials. A total of 366 patients randomized (2:1) to receive either IMGN853 (6 mg/kg) or single-agent chemotherapy (pegylated liposomal doxorubicin, topotecan, or paclitaxel). The primary endpoint of this study was PFS.

In early 2019, the published results showed no significant difference in the primary endpoint PFS [HR, 0.98; 95% CI, 0.73–1.31; *p* = 0.897] between groups, and the median PFS of IMGN853 group and chemotherapy group were 4.1 and 4.4 months, respectively.^240^ Although the primary endpoint was not reached, a better performance of IMGN853 was observed among the patients with a overexpressed FRα in secondary endpoints, including better ORR (24% vs. 10%) and CA-125 response (53% vs. 25%). Therefore, additional phase 3 trials were recommended by the FDA. In December 2019, Immunogen announced a phase 3 single-arm trial, which included two studies: SORAYA Study (NCT04296890) and MIRASOL Study (NCT04209855). Besides, another phase Ib/II clinical trial (NCT02606305) investigated the application of IMGN853 (6 mg/kg) combined with bevacizumab (15 mg/kg) in platinum resistant patients with advanced epithelial ovarian cancer, primary peritoneal cancer or fallopian tube cancer.^241^ Immunogen recently announced the results at the 2021 ASCO annual meeting. Among 60 patients received the combination, 28 were observed objective responses for a confirmed ORR of 47% (95% CI, 34–60), and in patients with high FRα expression (*n* = 33), the confirmed ORR was 64% (95% CI 45–80), which suggested a significant therapeutic effect of IMGN853 combination with bevacizumab for patients with high FRα recurrent ovarian cancer. The most common AEs included diarrhea, blurred vision, fatigue, and nausea.^242,243^

#### Datopotamab deruxtecan (DS-1062, Daiichi Sankyo/AstraZeneca)

DS-1062 or Dato-DXd, is the second Trop2-targeting ADC comprising of a topoisomerase I inhibitor (DXd) conjugated to a humanized anti-Trop2 IgG1 mAb through a cleavable tetrapeptide-based linker.^244^ The average DAR is 4. At present, DS-1062 is under evaluation for solid tumors like breast cancer and NSCLC using single or combination therapies in clinical trials. The updated results in the TROPION-PanTumor01, an ongoing phase 1 study in patients with advanced/ metastatic NSCLC (NCT03401385), showed a promising safety and efficacy in the patients received with DS-1062 (6 mg/kg).^245,246^ In 125 response-evaluable patients, 1% (1/125) had a confirmed CR, 26% (32/125) had PR and 4 PRs were awaiting confirmation. The probability of having an ongoing response at 6 months was over 80% and the disease control rate (CR + PR + SD) was 79%. It is also currently under investigation in a randomized, open-label, phase 3 study (TROPION-Lung01) for the comparison of DS-1062 *v.s*. docetaxel for the treatment of patients with advanced/metastatic NSCLC without EGFR, ALK, or other actionable genomic alterations.^247^ It is designed that 590 patients randomized 1:1 to receive either DS-1062 (6 mg/kg) or docetaxel (75 mg/m^2^). Dual primary endpoints are PFS and OS are used as dual primary endpoints.

#### Tusamitamab ravtansine (SAR-408701, Sanofi)

SAR-408701 is novel anti-CEACAM5 ADC. It is composed of a humanized antibody targeting CEACAM5 coupled to a cytotoxic maytansinoid DM4 via a cleavable linker N-succinimidyl 4-(2-pyridyldithio) butyrate (SPDB). CEACAM5 is glycoprotein that rarely expressed in normal adult tissues, but overexpressed in multiple solid tumors, including NSCLC.^248^ The preclinical activity of SAR-408701 was investigated and results showed a promising potential therapeutic candidate for CEACAM5 positive cancer.^249^ The safety and efficacy of SAR-408701 was examined in an open-label, dose-escalation, dose-expansion phase 1 study (NCT02187848). In the study, among 92 patients (28 moderate and 64 high expressors of CEACAM5) received with SAR-408701 (100 mg/m^2^), as of January 2020, 2 confirmed PR were observed (ORR 7.1%) in the moderate expressor cohort, while 13 patients had confirmed PRs (ORR 20.3%) in the high expressor cohort. 27 (42.2%) had stable disease; ORR of 17.8% was observed in the patients who previously received with anti-PD1/PD-L1.^250^ Currently, several phase 2 or phase 3 clinical trials of SAR-408701 are being carried out using single drug or combination therapy for NSCLC and other solid tumor indications (NCT04154956, NCT04659603, NCT04394624, NCT05071053, NCT04524689).

## Current challenges and next generations of ADCs

From the approved drugs and in development candidates listed in previous sections, it can be seen that new generation ADCs are demonstrating increasingly optimal specificity and cytotoxicity profiles than early generations. Nevertheless, there remain many challenges in the development of use of anti-cancer ADCs, including complexity in pharmacokinetics, insufficient tumor targeting and payload release, as well as drug resistance.^26^ This section provides an overview on these challenges, followed by discussions on potential solutions in emerging generation ADCs.

### Major challenges

#### Complex pharmacokinetic profiles

After administration (mostly via i.v. infusion) of an ADC, three main forms may be present in the systemic circulation, i.e., the intact ADC, the naked antibody, and the free cytotoxic payload.^251^ Because of target binding, elimination, and deconjugation, the proportions of these three forms will change dynamically.^129^ In a typical pharmacokinetic profile of ADC, the concentration of both conjugated ADC and naked antibody continues to decrease as the internalization of ADC and antibody clearance.^252^ Factors affecting antibody clearance include mononuclear phagocyte system and neonatal Fc receptor (FcRn)-mediated recycling.^37,253^ Through binding with ADC in endocytic vacuole, FcRn exports ADC to extracellular compartment for the recycling.^254^ Therefore, antibodies including conjugated ADC and naked antibody usually have a longer half-life compared with conventional small-molecule drugs. With regards to free cytotoxic payload, it is mainly metabolized in the liver and excreted from the body through the kidneys (urine) or in the feces, which could be affected by drug–drug interactions and damaged liver and kidney functions.^255^ All of these factors, combined with high interpatient variability, it is challenging to establish PK and PD models to describe clinical characteristics of ADC and to assist the design of new ADCs.

#### Unavoidable side effect

Among approved 14 ADCs, the most common severe side effect (grade 3 or higher) is hematotoxicity including neutropenia, thrombocytopenia, leukopenia, and anemia. The hematotoxicity along with hepatotoxicity, and gastrointestinal reaction are probably related with premature release of cytotoxic payloads into blood circulation.^254^ It is consistent with conventional chemotherapy drugs that mainly affect those rapidly proliferating healthy cells. Moreover, the immune response induced by the antibody part of ADC may cause secondary injuries, resulting in nephrotoxicity.^256^ According to the recent clinical observations, the potential lung toxic effects like ILD during ADC treatment period should arouse attention, particularly in anti-HER2 ADCs.^257,258^ Several death cases have been reported to be related with ILD during the clinical trials of T-DM1 and DS-8201.^257,258^ The detailed mechanism of action of ILD, however, remain unclear. There is speculation one of the possible reasons might be associated with the undesirable uptake of the ADC in healthy lung cells and free payload released from ADC.^259^ Because of the most abundant blood flow and the longest retention time in lung, the undesirable uptake of the ADC and the free payloads in blood most occurred is in lung to induce ILD.^260^ Therefore, the corresponding optimizations of next generations of ADC are required to minimize side effects. And during medication, the adverse reactions should be closely monitored, prevented or given supportive treatments.

#### Tumor targeting and payload release

Compared with traditional cytotoxic drugs, the molecular weight of ADC is much bigger that the efficiency of drug penetrating into tumors is limited. The current research shows that only a small part of ADC input into patients can reach tumor cells, thus the potency of payloads needs to be considered when designing ADC.^261^ For ADC drugs, the delivery of payloads depends on the internalization of formed ADC-antigen complex through antigen-dependent endocytosis or antigen-independent pinocytosis. After internalization, ADC antigen complex will be transported to endosome or lysosome for the release payloads. When payload connected by acid cleavable linker, it is likely to be released in the early endosome for those ADCs required specific proteases, the release of payload will be occurred in late endosomes or lysosomes.^262^ Regardless of the payload release pathway, some ADCs have a “bystander effect” that can affect surrounding cancer cells without expression of target antigens. For the internalized ADC, it is considered to be an important factor in the tumor activity of ADC with high heterogeneity of target antigen expression. The bystander effect requires the payload to cross the cell membrane, hence the non-polar payloads released by cleavable linker are preferred as polar molecules are more likely to remain in cells.^263^

#### Drug resistance

Another challenge for ADC development is the drug resistance. The drug resistance to tyrosine kinase inhibitor (TKI) usually involves the escape mutation of drug target.^264^ The mechanism of drug resistance of ADCs, however, have not been sufficiently characterized. They are likely more complex and diverse due to the underlying MOA of ADCs. The current evidence shows that tumors can escape the activity of ADC in many ways, such as reducing the expression level of antigen, changing intracellular transport pathway, drug resistance to payloads.^265^ These potential mechanisms have been verified in preclinical in vitro and animal studies, and the clinical evidence to confirm these mechanisms is still limited. For example, long-term exposure to HER2-targeting ADC, the breast cancer cell lines will reduce the expression of HER2 receptors and reduce lysosomal acidification to slow down protein degradation and metabolism.^266^ Some ATP-binding cassette (ABC) transporters have been found to be important in the export anticancer drugs and render tumors resistant.^267^ The common payloads used in ADC, like MMAE, MMAF and calicheamicin, can be exported outside cancer cells by ABC transporters, which makes these ADCs show drug resistance.^268^

### Future generation ADCs

ADC consists of monoclonal antibody, linker and payload, thus replacing any of these three components may affect the effectiveness of ADC. Different antibodies targeting the same antigen may have different binding abilities and have different effects on receptor dimerization and antigen internalization. Current studies have shown that ADC internalization and intracellular transport pathway have a key impact on the cytotoxic activity of ADC.^37^ Compared with wild-type proteins, mutant proteins usually have higher ubiquitination level and are easier to be internalized and degraded.^269^ It means that if ADC is used to target mutant proteins, it may bring significant clinical response. It is conceivable that targeting ADCs carrying oncogenic mutant proteins (such as some EGFR mutants) may maximize the tumor specificity of therapy and reach the level of selective TKI.

Moreover, the progress of bispecific antibody technology has brought more possibilities for ADC innovation. These ADC designs may improve antibody internalization and improve tumor specificity. Current therapies under development have been exploring these possibilities. For example, bispecific ADC targeting different sites on the same antigen can improve receptor aggregation and lead to rapid internalization of the target.^270^ In addition, a bispecific ADC dual-targeting HER2 and LAMP-3 showed better lysosomal aggregation and load delivery in preclinical experiments.^271^ Similarly, the dual-payload ADC that employs two distinct cytotoxic agents as payloads could be developed to reduce the drug resistance. Through accurately controlling the proportion of the two agents, a more potent efficacy could be achieved by the delivery of two synergetic payloads into cancer cells.^272^ And with the application of two payloads with different mechanisms, the incidence of drug resistance would be significantly reduced. For example, a homogeneous anti-HER2 ADC containing both MMAE and MMAF was designed and exerted more remarkable antitumor activity in xenograft mouse models than co-administration of corresponding single-payload ADCs.^273^

Another ADC development strategy is to abandon the traditional structure of mAb and choose to couple the payload to the polypeptide fragment or single chain variable region fragment with smaller molecular weight. The main purpose of these strategies is to reduce the molecular weight of ADCs, so as to improve the penetration efficiency and payload delivery to tumor tissues. For example, PEN-221 is a ADC comprising of DM-1 conjugated to a polypeptide chain targeting somatostatin receptor 2. Its molecular weight is only 2 kDa, far less than 150 kDa of IgG molecules in traditional ADC.^274^ The current technical challenge for such ADCs is that they may be rapidly cleared in plasma. However, if we can overcome this obstacle, it can have potential in the treatment of inaccessible tumors, including tumors with poor vascular innervation and central nervous system tumors. Classically, a high internalizing capacity of mAb is required ADC for the delivery of payloads into cancer cells. However, mAb is often difficult to diffuse into the solid tumor mass due to antigen barrier. Thus, non-internalizing antibody could be developed for ADC. It is based on the principle that payloads directly release extracellularly under reducing condition in tumor microenvironment and then diffuses inside the cancer cells resulting in cell death.^275^

Lastly, there are still a lot of innovative opportunities in payload selection. At present, the choice of loading is no longer limited to standard cytotoxic drugs, but began to discover more targeted drugs and immune drugs. For example, mirzotamab clezutoclax is an ADC targeting B7-H3, which employs novel BCL-XL inhibitors that promote cell apoptosis as payload. It is currently being evaluated in early clinical trials.^276^

## Conclusions

Decades of efforts from the academia and the industry have led to successful development of a variety of ADC therapies that benefit tens of thousands cancer patients. The launch of 14 ADC drugs and the exciting clinical performance of other ADC candidates have also been attracting more attention into the field, which is important for this relatively young, but highly complicated area. Fortunately,. a large number of studies have provided insights to the key elements that dictate the ultimate behavior of ADCs. It would be crucial to establish appropriate methods for the evaluation of each components of ADC in vitro and in vivo. Identification and validation of new antigen/antibodies, development of new payloads with optimal toxicity, and design of new linkers to balance between stability and payload release, appear to be critical for the next generation of ADCs. With the continuous efforts by researchers in these fields, it is not difficult to envisage that future ADCs will show more surprises in targeted therapy for cancer.

## References

[1] Sung H (2021). Global cancer statistics 2020: GLOBOCAN estimates of incidence and mortality worldwide for 36 cancers in 185 countries. CA Cancer J. Clin..

[2] Loadman P (2002). Anticancer drug development. Br. J. Cancer.

[3] Gilman A, Philips FS (1946). The biological actions and therapeutic applications of the B-chloroethyl amines and sulfides. Science.

[4] Heidelberger C (1957). Fluorinated pyrimidines, a new class of tumour-inhibitory compounds. Nature.

[5] Norris RE, Adamson PC (2010). Clinical potency of methotrexate, aminopterin, talotrexin and pemetrexed in childhood leukemias. Cancer Chemother. Pharmacol..

[6] Rosenberg B, Van Camp L, Krigas T (1965). Inhibition of cell division in Escherichia coli by electrolysis products from a platinum electrode. Nature.

[7] Rowinsky EK, Donehower RC (1995). Paclitaxel (taxol). N. Engl. J. Med..

[8] Lindley C (1999). Perception of chemotherapy side effects cancer versus noncancer patients. Cancer Pract..

[9] Strebhardt K, Ullrich A (2008). Paul Ehrlich’s magic bullet concept: 100 years of progress. Nat. Rev. Cancer.

[10] Iqbal N, Iqbal N (2014). Human epidermal growth factor receptor 2 (HER2) in cancers: overexpression and therapeutic implications. Mol. Biol. Int.

[11] Prevodnik VK, Lavrenčak J, Horvat M, Novakovič BJ (2011). The predictive significance of CD20 expression in B-cell lymphomas. Diagn. Pathol..

[12] Köhler G, Milstein C (1975). Continuous cultures of fused cells secreting antibody of predefined specificity. Nature.

[13] Ferrara N, Hillan KJ, Novotny W (2005). Bevacizumab (Avastin), a humanized anti-VEGF monoclonal antibody for cancer therapy. Biochem. Biophys. Res. Commun..

[14] McKeage K, Perry CM (2002). Trastuzumab. Drugs.

[15] Plosker GL, Figgitt DP (2003). Rituximab. Drugs.

[16] Blick SK, Scott LJ (2007). Cetuximab. Drugs.

[17] Shefet-Carasso L, Benhar I (2015). Antibody-targeted drugs and drug resistance—challenges and solutions. Drug Resist. Updat..

[18] Sievers EL, Senter PD (2013). Antibody-drug conjugates in cancer therapy. Annu. Rev. Med.

[19] Lambert JM, Berkenblit A (2018). Antibody–drug conjugates for cancer treatment. Annu. Rev. Med..

[20] Norsworthy KJ (2018). FDA approval summary: mylotarg for treatment of patients with relapsed or refractory CD33‐positive acute myeloid leukemia. Oncologist.

[21] Ethan Ennals For The Mail On Sunday. New breed of drug which reduces bladder cancer deaths could replace chemotherapy in other cases. At dailymail.co.uk (2021).

[22] Damelin M, Zhong W, Myers J, Sapra P (2015). Evolving strategies for target selection for antibody-drug conjugates. Pharm. Res..

[23] Diamantis N, Banerji U (2016). Antibody-drug conjugates—an emerging class of cancer treatment. Br. J. Cancer.

[24] Ritchie M, Tchistiakova L, Scott N (2013). Implications of receptor-mediated endocytosis and intracellular trafficking dynamics in the development of antibody drug conjugates. MAbs.

[25] Donaghy H (2016). Effects of antibody, drug and linker on the preclinical and clinical toxicities of antibody-drug conjugates. MAbs.

[26] Beck A, Goetsch L, Dumontet C, Corvaïa N (2017). Strategies and challenges for the next generation of antibody–drug conjugates. Nat. Rev. Drug Discov..

[27] Xiao Y, Yu D (2020). Tumor microenvironment as a therapeutic target in cancer. Pharmacol. Ther.

[28] Rummel S (2012). Genomic (in) stability of the breast tumor microenvironment. Mol. Cancer Res..

[29] De Cecco M, Galbraith DN, McDermott LL (2021). What makes a good antibody-drug conjugate?. Expert Opin. Biol. Ther..

[30] Hock MB, Thudium KE, Carrasco-Triguero M, Schwabe NF (2015). Immunogenicity of antibody drug conjugates: bioanalytical methods and monitoring strategy for a novel therapeutic modality. AAPS J..

[31] Abdollahpour‐Alitappeh M (2019). Antibody–drug conjugates (ADCs) for cancer therapy: Strategies, challenges, and successes. J. Cell. Physiol..

[32] Natsume A, Niwa R, Satoh M (2009). Improving effector functions of antibodies for cancer treatment: enhancing ADCC and CDC. Drug Des. Devel. Ther..

[33] Stapleton NM (2011). Competition for FcRn-mediated transport gives rise to short half-life of human IgG3 and offers therapeutic potential. Nat. Commun..

[34] Zhang J (2018). Structural changes and aggregation mechanisms of two different dimers of an IgG2 monoclonal antibody. Biochemistry.

[35] Spiess C (2013). Development of a human IgG4 bispecific antibody for dual targeting of interleukin-4 (IL-4) and interleukin-13 (IL-13) cytokines. J. Biol. Chem..

[36] Rispens T, Ooijevaar-de Heer P, Bende O, Aalberse RC (2011). Mechanism of immunoglobulin G4 Fab-arm exchange. J. Am. Chem. Soc..

[37] Xu S (2015). Internalization, trafficking, intracellular processing and actions of antibody-drug conjugates. Pharm. Res..

[38] Singh AP (2020). Antibody coadministration as a strategy to overcome binding-site barrier for ADCs: a quantitative investigation. AAPS J..

[39] Tsumura R (2018). Influence of the dissociation rate constant on the intra-tumor distribution of antibody-drug conjugate against tissue factor. J. Control. Release.

[40] Saunders KO (2019). Conceptual approaches to modulating antibody effector functions and circulation half-life. Front. Immunol..

[41] Bargh JD, Isidro-Llobet A, Parker JS, Spring DR (2019). Cleavable linkers in antibody–drug conjugates. Chem. Soc. Rev..

[42] Nolting B (2013). Linker technologies for antibody-drug conjugates. Methods Mol. Biol.

[43] Flygare JA, Pillow TH, Aristoff P (2013). Antibody‐drug conjugates for the treatment of cancer. Chem. Biol. Drug Des..

[44] Zhang D (2019). Catalytic cleavage of disulfide bonds in small molecules and linkers of antibody–drug conjugates. Drug Metab. Disposition.

[45] Pallardó FV, Markovic J, García JL, Viña J (2009). Role of nuclear glutathione as a key regulator of cell proliferation. Mol. Asp. Med..

[46] Estrela JM, Ortega A, Obrador E (2006). Glutathione in cancer biology and therapy. Crit. Rev. Clin. Lab. Sci..

[47] Doronina SO (2008). Novel peptide linkers for highly potent antibody− auristatin conjugate. Bioconjug. Chem..

[48] Gondi CS, Rao JS (2013). Cathepsin B as a cancer target. Expert Opin. Ther. Targets.

[49] Dubowchik GM (2002). Cathepsin B-labile dipeptide linkers for lysosomal release of doxorubicin from internalizing immunoconjugates: model studies of enzymatic drug release and antigen-specific in vitro anticancer activity. Bioconjug. Chem..

[50] Jeffrey SC (2007). Minor groove binder antibody conjugates employing a water soluble β-glucuronide linker. Bioorg. Med. Chem. Lett..

[51] Kovtun YV (2006). Antibody-drug conjugates designed to eradicate tumors with homogeneous and heterogeneous expression of the target antigen. Cancer Res..

[52] Oflazoglu E (2008). Potent anticarcinoma activity of the humanized anti-CD70 antibody h1F6 conjugated to the tubulin inhibitor auristatin via an uncleavable linker. Clin. Cancer Res..

[53] Erickson HK (2010). Tumor delivery and in vivo processing of disulfide-linked and thioether-linked antibody− maytansinoid conjugates. Bioconjug. Chem..

[54] Girish S (2012). Clinical pharmacology of trastuzumab emtansine (T-DM1): an antibody–drug conjugate in development for the treatment of HER2-positive cancer. Cancer Chemother. Pharmacol..

[55] Phillips GDL (2008). Targeting HER2-positive breast cancer with trastuzumab-DM1, an antibody–cytotoxic drug conjugate. Cancer Res..

[56] Zhao P (2020). Recent advances of antibody drug conjugates for clinical applications. Acta Pharm. Sin. B.

[57] Birrer MJ, Moore KN, Betella I, Bates RC (2019). Antibody-drug conjugate-based therapeutics: state of the science. J. Natl Cancer Inst..

[58] Yang H, Ganguly A, Cabral F (2010). Inhibition of cell migration and cell division correlates with distinct effects of microtubule inhibiting drugs. J. Biol. Chem..

[59] Kaur R (2014). Recent developments in tubulin polymerization inhibitors: an overview. Eur. J. Med. Chem..

[60] Walczak CE (2000). Microtubule dynamics and tubulin interacting proteins. Curr. Opin. Cell Biol..

[61] Koga Y (2015). Antitumor effect of antitissue factor antibody‐MMAE conjugate in human pancreatic tumor xenografts. Int. J. Cancer.

[62] Yao X (2015). A novel humanized anti-HER2 antibody conjugated with MMAE exerts potent anti-tumor activity. Breast Cancer Res. Treat..

[63] Lopus M (2011). Antibody-DM1 conjugates as cancer therapeutics. Cancer Lett..

[64] Sandmann A, Sasse F, Müller R (2004). Identification and analysis of the core biosynthetic machinery of tubulysin, a potent cytotoxin with potential anticancer activity. Chem. Biol..

[65] Morris M (2017). Phase 1 study of the PSMA-targeted small-molecule drug conjugate EC1169 in patients with metastatic castrate-resistant prostate cancer (mCRPC). Ann. Oncol..

[66] Cheung-Ong K, Giaever G, Nislow C (2013). DNA-damaging agents in cancer chemotherapy: serendipity and chemical biology. Chem. Biol..

[67] Elmroth K (2003). Cleavage of cellular DNA by calicheamicin γ1. DNA Repair.

[68] Boger DL (1995). The duocarmycins: synthetic and mechanistic studies. Acc. Chem. Res..

[69] Pommier Y (2009). DNA topoisomerase I inhibitors: chemistry, biology, and interfacial inhibition. Chem. Rev..

[70] Gregson SJ (2001). Design, synthesis, and evaluation of a novel pyrrolobenzodiazepine DNA-interactive agent with highly efficient cross-linking ability and potent cytotoxicity. J. Med. Chem..

[71] Zein N, Sinha AM, McGahren WJ, Ellestad GA (1988). Calicheamicin gamma 1I: an antitumor antibiotic that cleaves double-stranded DNA site specifically. Science.

[72] Takahashi I (1988). Duocarmycin A, a new antitumor antibiotic from Streptomyces. J. Antibiot..

[73] Kawato Y (1991). Intracellular roles of SN-38, a metabolite of the camptothecin derivative CPT-11, in the antitumor effect of CPT-11. Cancer Res..

[74] Meddahi A (1996). FGF protection and inhibition of human neutrophil elastase by carboxymethyl benzylamide sulfonate dextran derivatives. Int. J. Biol. Macromol..

[75] Gregson SJ (2017). Pyrrolobenzodiazepine dimer antibody–drug conjugates: synthesis and evaluation of noncleavable drug-linkers. J. Med. Chem..

[76] Kamal A (2002). Design, synthesis, and evaluation of new noncross-linking pyrrolobenzodiazepine dimers with efficient DNA binding ability and potent antitumor activity. J. Med. Chem..

[77] Lee A (2021). Loncastuximab tesirine: first approval. Drugs.

[78] Ackerman SE (2021). Immune-stimulating antibody conjugates elicit robust myeloid activation and durable antitumor immunity. Nat. Cancer.

[79] Qian C, Prieto J (2004). Gene therapy of cancer: induction of anti-tumor immunity. Cell. Mol. Immunol..

[80] He L (2021). Immune modulating antibody–drug conjugate (IM-ADC) for cancer immunotherapy. J. Med. Chem..

[81] Bukhalid RA (2020). Systemic administration of STING agonist antibody-drug conjugates elicit potent anti-tumor immune responses with minimal induction of circulating cytokines. Cancer Res..

[82] Medzhitov R (2001). Toll-like receptors and innate immunity. Nat. Rev. Immunol..

[83] Dumbrava EI (2021). Abstract OT-03-02: Phase 1/2 study of a novel HER2 targeting TLR7/8 immune-stimulating antibody conjugate (ISAC), BDC-1001, as a single agent and in combination with an immune checkpoint inhibitor in patients with advanced HER2-expressing solid tumors. Cancer Res..

[84] Metz H (2020). SBT6050, a HER2-directed TLR8 therapeutic, as a systemically administered, tumor-targeted human myeloid cell agonist. J. Clin. Oncol..

[85] Shi F (2021). Activation of STING inhibits cervical cancer tumor growth through enhancing the anti-tumor immune response. Mol. Cell Biochem..

[86] Amouzegar A (2021). STING agonists as cancer therapeutics. Cancers (Basel).

[87] Duvall JR (2021). XMT-2056, a well-tolerated, Immunosynthen-based STING-agonist antibody-drug conjugate which induces anti-tumor immune activity. Cancer Res..

[88] Mallet W (2021). 784 BDC-2034: discovery of a CEA-targeting immune-stimulating antibody conjugate (ISAC) for solid tumors. J. Immunother. Cancer.

[89] Sharma M (2021). 164P Preliminary results from a phase I/II study of BDC-1001, a novel HER2 targeting TLR7/8 immune-stimulating antibody conjugate (ISAC), in patients (pts) with advanced HER2-expressing solid tumors. Ann. Oncol..

[90] Brun M-P, Gauzy-Lazo L (2013). Protocols for lysine conjugation. Methods Mol. Biol.

[91] Matsuda Y, Mendelsohn BA (2021). An overview of process development for antibody-drug conjugates produced by chemical conjugation technology. Expert Opin. Biol. Ther..

[92] Fukunaga A (2018). Improvement of antibody affinity by introduction of basic amino acid residues into the framework region. Biochem. Biophys. Rep..

[93] Hagihara Y, Saerens D (2014). Engineering disulfide bonds within an antibody. BBA-Proteins Proteom..

[94] Gordon MR (2015). Field guide to challenges and opportunities in antibody–drug conjugates for chemists. Bioconjug. Chem..

[95] Nadkarni DV (2020). Conjugations to endogenous cysteine residues. Methods Mol. Biol.

[96] Levengood MR (2017). Orthogonal cysteine protection enables homogeneous multi‐drug antibody–drug conjugates. Angew. Chem. Int. Ed..

[97] Strop P (2013). Location matters: site of conjugation modulates stability and pharmacokinetics of antibody drug conjugates. Chem. Biol..

[98] Shen B-Q (2012). Conjugation site modulates the in vivo stability and therapeutic activity of antibody-drug conjugates. Nat. Biotechnol..

[99] Junutula JR (2008). Site-specific conjugation of a cytotoxic drug to an antibody improves the therapeutic index. Nat. Biotechnol..

[100] Junutula JR (2010). Engineered thio-trastuzumab-DM1 conjugate with an improved therapeutic index to target human epidermal growth factor receptor 2–positive breast cancer. Clin. Cancer Res..

[101] Kung Sutherland MS (2013). SGN-CD33A: a novel CD33-targeting antibody–drug conjugate using a pyrrolobenzodiazepine dimer is active in models of drug-resistant AML. Blood.

[102] Liberatore FA (1990). Site-directed chemical modification and crosslinking of a monoclonal antibody using equilibrium transfer alkylating crosslink reagents. Bioconjug. Chem..

[103] Smith ME (2010). Protein modification, bioconjugation, and disulfide bridging using bromomaleimides. J. Am. Chem. Soc..

[104] Chudasama V (2011). Bromopyridazinedione-mediated protein and peptide bioconjugation. Chem. Commun..

[105] Schumacher FF (2014). Next generation maleimides enable the controlled assembly of antibody–drug conjugates via native disulfide bond bridging. Org. Biomol. Chem..

[106] Morais M, Forte N, Chudasama V, Baker JR (2019). Application of next-generation maleimides (NGMs) to site-selective antibody conjugation. Bioconjugation.

[107] Forte N, Chudasama V, Baker JR (2018). Homogeneous antibody-drug conjugates via site-selective disulfide bridging. Drug Discov. Today.: Technol..

[108] Hallam TJ, Wold E, Wahl A, Smider VV (2015). Antibody conjugates with unnatural amino acids. Mol. Pharm..

[109] Zhou Q (2017). Site-specific antibody conjugation for ADC and beyond. Biomedicines.

[110] Hallam TJ, Smider VV (2014). Unnatural amino acids in novel antibody conjugates. Future Med. Chem..

[111] Rao C, Rangan VS, Deshpande S (2015). Challenges in antibody–drug conjugate discovery: a bioconjugation and analytical perspective. Bioanalysis.

[112] Kim EG, Kim KM (2015). Strategies and advancement in antibody-drug conjugate optimization for targeted cancer therapeutics. Biomol. Ther..

[113] Agarwal P, Bertozzi CR (2015). Site-specific antibody–drug conjugates: the nexus of bioorthogonal chemistry, protein engineering, and drug development. Bioconjug. Chem..

[114] Subedi GP, Barb AW (2015). The structural role of antibody N-glycosylation in receptor interactions. Structure.

[115] Walsh SJ (2021). Site-selective modification strategies in antibody–drug conjugates. Chem. Soc. Rev..

[116] Cao YJ (2021). Synthesis of precision antibody conjugates using proximity-induced chemistry. Theranostics.

[117] Adair JR (2012). Antibody–drug conjugates–a perfect synergy. Expert Opin. Biol. Ther..

[118] Staudacher AH, Brown MP (2017). Antibody drug conjugates and bystander killing: is antigen-dependent internalisation required?. Br. J. Cancer.

[119] Tai Y-T (2014). Novel anti–B-cell maturation antigen antibody-drug conjugate (GSK2857916) selectively induces killing of multiple myeloma. Blood.

[120] Radocha J, van de Donk NW, Weisel K (2021). Monoclonal antibodies and antibody drug conjugates in multiple myeloma. Cancers (Basel).

[121] Oostra DR, Macrae ER (2014). Role of trastuzumab emtansine in the treatment of HER2-positive breast cancer. Breast Cancer (Lond.).

[122] Dosio F, Brusa P, Cattel L (2011). Immunotoxins and anticancer drug conjugate assemblies: the role of the linkage between components. Toxins (Basel).

[123] Chari RV (1998). Targeted delivery of chemotherapeutics: tumor-activated prodrug therapy. Adv. Drug Del. Rev..

[124] McGavin JK, Spencer CM (2001). Gemtuzumab ozogamicin. Drugs.

[125] Lamb YN (2017). Inotuzumab ozogamicin: first global approval. Drugs.

[126] Hinman LM (1993). Preparation and characterization of monoclonal antibody conjugates of the calicheamicins: a novel and potent family of antitumor antibiotics. Cancer Res..

[127] Kaytor MD, Wilkinson KD, Warren ST (2004). Modulating huntingtin half‐life alters polyglutamine‐dependent aggregate formation and cell toxicity. J. Neurochem..

[128] Siegel MM (1997). Calicheamicin derivatives conjugated to monoclonal antibodies: determination of loading values and distributions by infrared and UV matrix-assisted laser desorption/ionization mass spectrometry and electrospray ionization mass spectrometry. Anal. Chem..

[129] Lucas AT (2018). Factors affecting the pharmacology of antibody–drug conjugates. Antibodies.

[130] Strop P (2015). Site-specific conjugation improves therapeutic index of antibody drug conjugates with high drug loading. Nat. Biotechnol..

[131] Kamath AV, Iyer S (2015). Preclinical pharmacokinetic considerations for the development of antibody drug conjugates. Pharm. Res..

[132] Katz J, Janik JE, Younes A (2011). Brentuximab vedotin (SGN-35). Clin. Cancer Res..

[133] Lambert JM, Chari RV (2014). Ado-trastuzumab Emtansine (T-DM1): an antibody–drug conjugate (ADC) for HER2-positive breast cancer. J. Medicinal Chem..

[134] Tsuchikama K, An Z (2018). Antibody-drug conjugates: recent advances in conjugation and linker chemistries. Protein Cell.

[135] Sun X (2017). Effects of drug–antibody ratio on pharmacokinetics, biodistribution, efficacy, and tolerability of antibody–maytansinoid conjugates. Bioconjug. Chem..

[136] Lyon RP (2015). Reducing hydrophobicity of homogeneous antibody-drug conjugates improves pharmacokinetics and therapeutic index. Nat. Biotechnol..

[137] Burke PJ (2017). Optimization of a PEGylated glucuronide-monomethylauristatin E linker for antibody–drug conjugates. Mol. Cancer Ther..

[138] Hoffmann RM (2018). Antibody structure and engineering considerations for the design and function of Antibody Drug Conjugates (ADCs). Oncoimmunology.

[139] Jäger S (2021). Generation and biological evaluation of Fc antigen binding fragment-drug conjugates as a novel antibody-based format for targeted drug delivery. Bioconjug. Chem..

[140] Yurkovetskiy AV (2015). A polymer-based antibody–vinca drug conjugate platform: characterization and preclinical efficacy. Cancer Res..

[141] Bodyak N (2015). Trastuzumab-dolaflexin, a highly potent Fleximer-based antibody-drug conjugate, demonstrates a favorable therapeutic index in exploratory toxicology studies in multiple species. Cancer Res..

[142] Simmons JK (2020). Reducing the antigen-independent toxicity of antibody-drug conjugates by minimizing their non-specific clearance through PEGylation. Toxicol. Appl. Pharmacol..

[143] Shao T (2020). Construction of paclitaxel-based antibody–drug conjugates with a PEGylated linker to achieve superior therapeutic index. Signal Transduct. Target. Ther..

[144] Buecheler JW (2018). Impact of payload hydrophobicity on the stability of antibody–drug conjugates. Mol. Pharm..

[145] Bross PF (2001). Approval summary: gemtuzumab ozogamicin in relapsed acute myeloid leukemia. Clin. Cancer Res..

[146] Guglielmi C (1998). Immunophenotype of adult and childhood acute promyelocytic leukaemia: correlation with morphology, type of PML gene breakpoint and clinical outcome. A cooperative Italian study on 196 cases. Br. J. Haematol..

[147] Parigger J, Zwaan C, Reinhardt D, Kaspers G (2016). Dose-related efficacy and toxicity of gemtuzumab ozogamicin in pediatric acute myeloid leukemia. Expert Rev. Anticancer Ther..

[148] Petersdorf S (2009). Preliminary results of Southwest Oncology Group study S0106: An international intergroup phase 3 randomized trial comparing the addition of Gemtuzumab ozogamicin to standard induction therapy versus standard induction therapy followed by a second randomization to post-consolidation Gemtuzumab ozogamicin versus no additional therapy for previously untreated acute myeloid leukemia. Blood.

[149] Castaigne S (2012). Effect of gemtuzumab ozogamicin on survival of adult patients with de-novo acute myeloid leukaemia (ALFA-0701): a randomised, open-label, phase 3 study. Lancet.

[150] Amadori S (2016). Gemtuzumab ozogamicin versus best supportive care in older patients with newly diagnosed acute myeloid leukemia unsuitable for intensive chemotherapy: results of the randomized phase III EORTC-GIMEMA AML-19 trial. J. Clin. Oncol..

[151] Burnett AK (2011). Identification of patients with acute myeloblastic leukemia who benefit from the addition of gemtuzumab ozogamicin: results of the MRC AML15 trial. J. Clin. Oncol..

[152] Hills RK (2014). Addition of gemtuzumab ozogamicin to induction chemotherapy in adult patients with acute myeloid leukaemia: a meta-analysis of individual patient data from randomised controlled trials. Lancet Oncol..

[153] Lamba JK (2017). CD33 splicing polymorphism determines gemtuzumab ozogamicin response in de novo acute myeloid leukemia: report from randomized phase III Children’s Oncology Group trial AAML0531. J. Clin. Oncol..

[154] Younes A, Yasothan U, Kirkpatrick P (2012). Brentuximab vedotin. Nat. Rev. Drug Discov..

[155] Hamblett KJ (2005). SGN-35, an anti-CD30 antibody-drug conjugate, exhibits potent antitumor activity for the treatment of CD30+ malignancies. Blood.

[156] Best RL (2021). Microtubule and tubulin binding and regulation of microtubule dynamics by the antibody drug conjugate (ADC) payload, monomethyl auristatin E (MMAE): Mechanistic insights into MMAE ADC peripheral neuropathy. Toxicol. Appl. Pharmacol..

[157] Bartlett NL (2013). A phase 2 study of brentuximab vedotin in patients with relapsed or refractory CD30-positive non-Hodgkin lymphomas: interim results in patients with DLBCL and other B-cell lymphomas. Blood.

[158] Pro B (2013). Three-year survival results from an ongoing phase 2 study of brentuximab vedotin in patients with relapsed or refractory systemic anaplastic large cell lymphoma. Blood.

[159] Chen R (2011). Results from a pivotal phase II study of brentuximab vedotin (SGN-35) in patients with relapsed or refractory Hodgkin lymphoma (HL). J. Clin. Oncol..

[160] Younes A (2012). Results of a pivotal phase II study of brentuximab vedotin for patients with relapsed or refractory Hodgkin’s lymphoma. J. Clin. Oncol..

[161] Pro B (2011). Durable remissions with brentuximab vedotin (SGN-35): updated results of a phase II study in patients with relapsed or refractory systemic anaplastic large cell lymphoma (sALCL). J. Clin. Oncol..

[162] Pro B (2012). Brentuximab vedotin (SGN-35) in patients with relapsed or refractory systemic anaplastic large-cell lymphoma: results of a phase II study. J. Clin. Oncol..

[163] Horwitz SM (2021). Randomized phase 3 ALCANZA study of brentuximab vedotin vs physician’s choice in cutaneous T-cell lymphoma: final data. Blood Adv.

[164] Richardson NC (2019). FDA approval summary: brentuximab vedotin in first‐line treatment of peripheral T‐Cell lymphoma. Oncologist.

[165] Straus DJ (2021). Brentuximab vedotin with chemotherapy for stage III or IV classical Hodgkin lymphoma (ECHELON-1): 5-year update of an international, open-label, randomised, phase 3 trial. Lancet Haematol..

[166] Shah NN (2015). Characterization of CD22 expression in acute lymphoblastic leukemia. Pediatr. Blood Cancer.

[167] Lanza F (2020). CD22 expression in b-cell acute lymphoblastic leukemia: biological significance and implications for inotuzumab therapy in adults. Cancers (Basel).

[168] Kantarjian HM (2019). Inotuzumab ozogamicin versus standard of care in relapsed or refractory acute lymphoblastic leukemia: Final report and long‐term survival follow‐up from the randomized, phase 3 INO‐VATE study. Cancer.

[169] Kantarjian HM (2016). Inotuzumab ozogamicin versus standard therapy for acute lymphoblastic leukemia. N. Engl. J. Med..

[170] Turner A, Kjeldsberg CR (1978). Hairy cell leukemia: a review. Medicine.

[171] Kreitman RJ, Pastan I (2011). Antibody fusion proteins: anti-CD22 recombinant immunotoxin moxetumomab pasudotox. Clin. Cancer Res..

[172] Cordone I (1995). Diagnostic relevance of peripheral blood immunocytochemistry in hairy cell leukaemia. J. Clin. Pathol..

[173] Babuŝíková O, Tomova A, Kusenda J, Gyarfas J (2001). Flow cytometry of peripheral blood and bone marrow cells from patients with hairy cell leukemia: phenotype of hairy cells, lymphocyte subsets and detection of minimal residual disease after treatment. Neoplasma.

[174] Janus A, Robak T (2019). Moxetumomab pasudotox for the treatment of hairy cell leukemia. Expert Opin. Biol. Ther..

[175] Kreitman RJ (2019). Moxetumomab Pasudotox-Tdfk in heavily pretreated patients with relapsed/refractory hairy cell leukemia (HCL): long-term follow-up from the pivotal Phase 3 Trial. Blood.

[176] Kreitman RJ (2021). Moxetumomab pasudotox in heavily pre-treated patients with relapsed/refractory hairy cell leukemia (HCL): long-term follow-up from the pivotal trial. J. Hematol. Oncol..

[177] Biocodex’s G (2018). FDA new drug approvals in Q3 2018. Nat. Rev. Drug Discov..

[178] Deeks ED (2019). Polatuzumab vedotin: first global approval. Drugs.

[179] Zheng B (2009). In vivo effects of targeting CD79b with antibodies and antibody-drug conjugates. Mol. Cancer Ther..

[180] Pfeifer M (2015). Anti-CD22 and anti-CD79B antibody drug conjugates are active in different molecular diffuse large B-cell lymphoma subtypes. Leukemia.

[181] Urquhart L (2019). FDA new drug approvals in Q2 2019. Nat. Rev. Drug Discov..

[182] Sehn LH (2020). Polatuzumab vedotin plus bendamustine and rituximab in relapsed/refractory diffuse large B-cell lymphoma: updated results of a phase Ib/II randomized study and preliminary results of a single-arm extension. Blood.

[183] Seckinger A (2017). Target expression, generation, preclinical activity, and pharmacokinetics of the BCMA-T cell bispecific antibody EM801 for multiple myeloma treatment. Cancer Cell.

[184] Lonial S (2020). Belantamab mafodotin for relapsed or refractory multiple myeloma (DREAMM-2): a two-arm, randomised, open-label, phase 2 study. Lancet Oncol..

[185] Jain N (2020). Loncastuximab tesirine, an anti-CD19 antibody-drug conjugate, in relapsed/refractory B-cell acute lymphoblastic leukemia. Blood Adv..

[186] Zammarchi F (2018). ADCT-402, a PBD dimer–containing antibody drug conjugate targeting CD19-expressing malignancies. Blood.

[187] Hartley JA (2021). Antibody-drug conjugates (ADCs) delivering pyrrolobenzodiazepine (PBD) dimers for cancer therapy. Expert Opin. Biol. Ther..

[188] Hartley JA (2011). The development of pyrrolobenzodiazepines as antitumour agents. Expert Opin. Investig. Drugs.

[189] Staben LR (2020). Systematic variation of pyrrolobenzodiazepine (PBD)-dimer payload physicochemical properties impacts efficacy and tolerability of the corresponding antibody–drug conjugates. J. Med. Chem..

[190] Caimi PF (2021). Loncastuximab tesirine in relapsed or refractory diffuse large B-cell lymphoma (LOTIS-2): a multicentre, open-label, single-arm, phase 2 trial. Lancet Oncol..

[191] Abraham J (2007). Trastuzumab emtansine in advanced HER2-positive breast cancer. Clin. Cancer Res..

[192] Slamon DJ (1987). Human breast cancer: correlation of relapse and survival with amplification of the HER-2/neu oncogene. Science.

[193] Junttila TT (2011). Trastuzumab-DM1 (T-DM1) retains all the mechanisms of action of trastuzumab and efficiently inhibits growth of lapatinib insensitive breast cancer. Breast Cancer Res. Treat..

[194] Diéras V (2017). Trastuzumab emtansine versus capecitabine plus lapatinib in patients with previously treated HER2-positive advanced breast cancer (EMILIA): a descriptive analysis of final overall survival results from a randomised, open-label, phase 3 trial. Lancet Oncol..

[195] Blackwell KL (2012). Primary results from EMILIA, a phase III study of trastuzumab emtansine (T-DM1) versus capecitabine (X) and lapatinib (L) in HER2-positive locally advanced or metastatic breast cancer (MBC) previously treated with trastuzumab (T) and a taxane. J. Clin. Oncol..

[196] Pondé N (2020). Trastuzumab emtansine (T-DM1)-associated cardiotoxicity: pooled analysis in advanced HER2-positive breast cancer. Eur. J. Cancer.

[197] Wedam S (2020). FDA Approval summary: ado-trastuzumab emtansine for the adjuvant treatment of HER2-positive early breast cancer. Clin. Cancer Res..

[198] Mamounas E (2021). Adjuvant T-DM1 versus trastuzumab in patients with residual invasive disease after neoadjuvant therapy for HER2-positive breast cancer: subgroup analyses from KATHERINE. Ann. Oncol..

[199] Chang E (2021). FDA approval summary: enfortumab vedotin for locally advanced or metastatic urothelial carcinoma. Clin. Cancer Res..

[200] Challita-Eid PM (2016). Enfortumab vedotin antibody–drug conjugate targeting nectin-4 is a highly potent therapeutic agent in multiple preclinical cancer models. Cancer Res..

[201] Liu Y (2021). Role of Nectin‑4 protein in cancer. Int. J. Oncol..

[202] Heath EI, Rosenberg JE (2021). The biology and rationale of targeting nectin-4 in urothelial carcinoma. Nat. Rev. Urol..

[203] Powles T (2021). Primary results of EV-301: A phase III trial of enfortumab vedotin versus chemotherapy in patients with previously treated locally advanced or metastatic urothelial carcinoma. J. Clin. Oncol..

[204] Petrylak DP (2019). EV-301: Phase III study to evaluate enfortumab vedotin (EV) versus chemotherapy in patients with previously treated locally advanced or metastatic urothelial cancer (la/mUC). J. Clin. Oncol..

[205] Evan YY (2021). Enfortumab vedotin after PD-1 or PD-L1 inhibitors in cisplatin-ineligible patients with advanced urothelial carcinoma (EV‑201): a multicentre, single-arm, phase 2 trial. Lancet Oncol..

[206] Shitara K (2021). Discovery and development of trastuzumab deruxtecan and safety management for patients with HER2-positive gastric cancer. Gastric Cancer.

[207] Modi S (2020). Trastuzumab deruxtecan in previously treated HER2-positive breast cancer. N. Engl. J. Med..

[208] Modi S (2021). Updated results from DESTINY-breast01, a phase 2 trial of trastuzumab deruxtecan (T-DXd) in HER2 positive metastatic breast cancer. Cancer Res..

[209] Cortés J (2021). LBA1 - Trastuzumab deruxtecan (T-DXd) vs trastuzumab emtansine (T-DM1) in patients (Pts) with HER2+ metastatic breast cancer (mBC): Results of the randomized phase III DESTINY-Breast03 study. Ann. Oncol..

[210] Cortés J (2021). LBA1 Trastuzumab deruxtecan (T-DXd) vs trastuzumab emtansine (T-DM1) in patients (Pts) with HER2+ metastatic breast cancer (mBC): Results of the randomized phase III DESTINY-Breast03 study. Ann. Oncol..

[211] Tolaney S (2021). 328TiP Phase III study of trastuzumab deruxtecan (T-DXd) with or without pertuzumab vs a taxane, trastuzumab and pertuzumab in first-line (1L), human epidermal growth factor receptor 2–positive (HER2+) metastatic breast cancer (mBC): DESTINY-Breast09. Ann. Oncol..

[212] Li BT (2022). Trastuzumab deruxtecan in HER2-mutant non–small-cell lung cancer. N. Engl. J. Med..

[213] Cottin V (2013). Interstitial lung disease. Eur. Respir. Rev..

[214] Janjigian Y (2020). 1500TiP A phase Ib/II, multicenter, open-label, dose-escalation and dose-expansion study evaluating trastuzumab deruxtecan (T-DXd; DS-8201) monotherapy and combinations in patients with HER2-overexpressing gastric cancer (DESTINY-Gastric03). Ann. Oncol..

[215] Lipinski M, Parks DR, Rouse RV, Herzenberg LA (1981). Human trophoblast cell-surface antigens defined by monoclonal antibodies. Proc. Natl Acad. Sci..

[216] Rapani E, Sacchetti A, Corda D, Alberti S (1998). Human Trop‐2 is a tumor‐associated calcium signal transducer. Int. J. Cancer.

[217] Wang J (2008). Identification of Trop-2 as an oncogene and an attractive therapeutic target in colon cancers. Mol. Cancer Ther..

[218] Zeng P (2016). Impact of TROP2 expression on prognosis in solid tumors: a systematic review and meta-analysis. Sci. Rep..

[219] Perrone E (2020). Sacituzumab govitecan, an antibody‐drug conjugate targeting trophoblast cell‐surface antigen 2, shows cytotoxic activity against poorly differentiated endometrial adenocarcinomas in vitro and in vivo. Mol. Oncol..

[220] Sahota S, Vahdat LT (2017). Sacituzumab govitecan: an antibody–drug conjugate. Expert Opin. Biol. Ther..

[221] Bardia A (2021). Sacituzumab govitecan in metastatic triple-negative breast cancer. N. Engl. J. Med..

[222] Bardia A (2020). LBA17 ASCENT: a randomized phase III study of sacituzumab govitecan (SG) vs treatment of physician’s choice (TPC) in patients (pts) with previously treated metastatic triple-negative breast cancer (mTNBC). Ann. Oncol..

[223] O’Shaughnessy J (2021). Assessment of sacituzumab govitecan (SG) versus treatment of physician’s choice (TPC) cohort by agent in the phase 3 ASCENT study of patients (pts) with metastatic triple-negative breast cancer (mTNBC). J. Clin. Oncol..

[224] Li J, Wang R, Gao J (2021). Novel anticancer drugs approved in 2020. Drug Discov. Ther.

[225] Kaplon H, Reichert JM (2021). Antibodies to watch in 2021. MAbs.

[226] Kitamura N (2021). Current trends and future prospects of molecular targeted therapy in head and neck squamous cell carcinoma. Int. J. Mol. Sci..

[227] Cognetti DM (2019). Results of a phase 2a, multicenter, open-label, study of RM-1929 photoimmunotherapy (PIT) in patients with locoregional, recurrent head and neck squamous cell carcinoma (rHNSCC). J. Clin. Oncol..

[228] Gillenwater AM (2018). RM-1929 photo-immunotherapy in patients with recurrent head and neck cancer: Results of a multicenter phase 2a open-label clinical trial. J. Clin. Oncol..

[229] Jiang J (2020). Preclinical safety profile of disitamab vedotin: a novel anti-HER2 antibody conjugated with MMAE. Toxicol. Lett..

[230] Xu Y (2021). Phase I study of the recombinant humanized anti-HER2 monoclonal antibody-MMAE conjugate RC48-ADC in patients with HER2-positive advanced solid tumors. Gastric Cancer.

[231] Peng Z (2020). A phase II study of efficacy and safety of RC48-ADC in patients with locally advanced or metastatic HER2-overexpressing gastric or gastroesophageal junction cancers. J. Clin. Oncol..

[232] Peng Z (2021). Efficacy and safety of a novel anti-HER2 therapeutic antibody RC48 in patients with HER2-overexpressing, locally advanced or metastatic gastric or gastroesophageal junction cancer: a single-arm phase II study. Cancer Commun..

[233] Sheng X (2021). An open-label, single-arm, multicenter, phase II study of RC48-ADC to evaluate the efficacy and safety of subjects with HER2 overexpressing locally advanced or metastatic urothelial cancer (RC48-C009). J. Clin. Oncol.

[234] Alley SC (2019). Tisotumab vedotin induces anti-tumor activity through MMAE-mediated, Fc-mediated, and Fab-mediated effector functions in vitro. Cancer Res..

[235] Liu Y (2011). Tissue factor–activated coagulation cascade in the tumor microenvironment is critical for tumor progression and an effective target for therapy. Cancer Res..

[236] Coleman RL (2021). Efficacy and safety of tisotumab vedotin in previously treated recurrent or metastatic cervical cancer (innovaTV 204/GOG-3023/ENGOT-cx6): a multicentre, open-label, single-arm, phase 2 study. Lancet Oncol..

[237] De Bono JS (2019). Tisotumab vedotin in patients with advanced or metastatic solid tumours (InnovaTV 201): a first-in-human, multicentre, phase 1–2 trial. Lancet Oncol..

[238] Ab O (2015). IMGN853, a folate receptor-α (FRα)–targeting antibody–drug conjugate, exhibits potent targeted antitumor activity against FRα-expressing tumors. Mol. Cancer Ther..

[239] Moore KN (2017). FORWARD I (GOG 3011): A randomized phase 3 study to evaluate the safety and efficacy of mirvetuximab soravtansine (IMGN853) versus chemotherapy in adults with folate receptor alpha (FRα)-positive, platinum-resistant epithelial ovarian cancer (EOC), primary peritoneal cancer, or primary fallopian tube cancer. J. Clin. Oncol..

[240] Moore K (2019). FORWARD I (GOG 3011): A phase III study of mirvetuximab soravtansine, a folate receptor alpha (FRa)-targeting antibody-drug conjugate (ADC), versus chemotherapy in patients (pts) with platinum-resistant ovarian cancer (PROC). Ann. Oncol..

[241] Moore KN (2017). Safety and activity of mirvetuximab soravtansine (IMGN853), a folate receptor alpha–targeting antibody–drug conjugate, in platinum-resistant ovarian, fallopian tube, or primary peritoneal cancer: a phase I expansion study. J. Clin. Oncol..

[242] O’Malley DM (2020). Phase Ib study of mirvetuximab soravtansine, a folate receptor alpha (FRα)-targeting antibody-drug conjugate (ADC), in combination with bevacizumab in patients with platinum-resistant ovarian cancer. Gynecol. Oncol..

[243] O’Malley DM (2021). Mirvetuximab soravtansine, a folate receptor alpha (FRα)-targeting antibody-drug conjugate (ADC), in combination with bevacizumab in patients (pts) with platinum-agnostic ovarian cancer: Final analysis. J. Clin. Oncol..

[244] Okajima D (2021). Datopotamab deruxtecan, a novel TROP2-directed antibody–drug conjugate, demonstrates potent antitumor activity by efficient drug delivery to tumor cells. Mol. Cancer Ther..

[245] Spira A (2021). OA03. 03 Datopotamab deruxtecan (Dato-DXd; DS-1062), a TROP2 ADC, in patients with advanced NSCLC: updated results of TROPION-PanTumor01 phase 1 study. J. Thorac. Oncol..

[246] Shimizu T (2021). O2-1 Datopotamab Deruxtecan (Dato-DXd; DS-1062), a TROP2 ADC, in patients with advanced NSCLC: Updated results of TROPION-PanTumor01 phase 1 study. Ann. Oncol..

[247] Yoh K (2021). A randomized, phase 3 study of datopotamab deruxtecan (Dato-DXd; DS-1062) versus docetaxel in previously treated advanced or metastatic non-small cell lung cancer (NSCLC) without actionable genomic alterations (TROPION-Lung01). J. Clin. Oncol..

[248] Zhang X (2020). CEACAM5 stimulates the progression of non-small-cell lung cancer by promoting cell proliferation and migration. J. Int. Med. Res..

[249] Decary S (2020). Preclinical activity of SAR408701: a novel anti-CEACAM5–maytansinoid antibody–drug conjugate for the treatment of CEACAM5-positive epithelial tumors. Clin. Cancer Res..

[250] Gazzah A (2020). Efficacy and safety of the antibody-drug conjugate (ADC) SAR408701 in patients (pts) with non-squamous non-small cell lung cancer (NSQ NSCLC) expressing carcinoembryonic antigen-related cell adhesion molecule 5 (CEACAM5). J. Clin. Oncol..

[251] Guo J (2016). Characterization and higher-order structure assessment of an interchain cysteine-based ADC: impact of drug loading and distribution on the mechanism of aggregation. Bioconjug. Chem..

[252] Malik P, Phipps C, Edginton A, Blay J (2017). Pharmacokinetic considerations for antibody-drug conjugates against cancer. Pharm. Res..

[253] Hamblett KJ (2016). Altering antibody–drug conjugate binding to the neonatal Fc receptor impacts efficacy and tolerability. Mol. Pharm..

[254] Mahalingaiah PK (2019). Potential mechanisms of target-independent uptake and toxicity of antibody-drug conjugates. Pharmacol. Ther..

[255] Khera E, Thurber GM (2018). Pharmacokinetic and immunological considerations for expanding the therapeutic window of next-generation antibody–drug conjugates. Biodrugs.

[256] Mecklenburg L (2018). A brief introduction to antibody–drug conjugates for toxicologic pathologists. Toxicol. Pathol..

[257] Hackshaw MD (2020). Incidence of pneumonitis/interstitial lung disease induced by HER2-targeting therapy for HER2-positive metastatic breast cancer. Breast Cancer Res. Treat..

[258] Powell C (2020). 289P Risk factors for interstitial lung disease in patients treated with trastuzumab deruxtecan from two interventional studies. Ann. Oncol..

[259] Tarantino P (2021). Interstitial lung disease induced by anti-ERBB2 antibody-drug conjugates: a review. JAMA Oncol..

[260] Spira A (2021). OA03.03 Datopotamab deruxtecan (Dato-DXd; DS-1062), a TROP2 ADC, in patients with advanced NSCLC: updated results of TROPION-PanTumor01 phase 1 study. J. Thorac. Oncol..

[261] Jin Y (2021). Stepping forward in antibody-drug conjugate development. Pharmacol. Ther.

[262] Tumey LN (2020). An Overview of the Current ADC Discovery Landscape. Antibody-Drug Conjugates.

[263] Singh AP, Shah DK (2019). A “dual” cell-level systems PK-PD model to characterize the bystander effect of ADC. J. Pharm. Sci..

[264] Wu S-G, Shih J-Y (2018). Management of acquired resistance to EGFR TKI–targeted therapy in advanced non-small cell lung cancer. Mol. Cancer.

[265] Loganzo F, Sung M, Gerber H-P (2016). Mechanisms of resistance to antibody–drug conjugates. Mol. Cancer Ther..

[266] Irie H (2020). Acquired resistance to trastuzumab/pertuzumab or to T‐DM1 in vivo can be overcome by HER2 kinase inhibition with TAS0728. Cancer Sci..

[267] Sipos G, Kuchler K (2006). Fungal ATP-binding cassette (ABC) transporters in drug resistance & detoxification. Curr. Drug Targets.

[268] Buongervino SN (2021). Antibody-drug conjugate efficacy in neuroblastoma-role of payload, resistance mechanisms, target density, and antibody internalization. Mol. Cancer Ther.

[269] Lee YT, Tan YJ, Oon CE (2018). Molecular targeted therapy: treating cancer with specificity. Eur. J. Pharmacol..

[270] Andreev J (2017). Bispecific antibodies and antibody–drug conjugates (ADCs) bridging HER2 and prolactin receptor improve efficacy of HER2 ADCs. Mol. Cancer Ther..

[271] de Goeij BE (2016). Efficient payload delivery by a bispecific antibody–drug conjugate targeting HER2 and CD63. Mol. Cancer Ther..

[272] Tang F (2016). One-pot N-glycosylation remodeling of IgG with non-natural sialylglycopeptides enables glycosite-specific and dual-payload antibody–drug conjugates. Org. Biomol. Chem..

[273] Yamazaki CM (2021). Antibody-drug conjugates with dual payloads for combating breast tumor heterogeneity and drug resistance. Nat. Commun..

[274] Whalen KA (2019). Targeting the somatostatin receptor 2 with the miniaturized drug conjugate, PEN-221: a potent and novel therapeutic for the treatment of small cell lung cancer. Mol. Cancer Ther..

[275] Dal Corso A (2017). A non-internalizing antibody-drug conjugate based on an anthracycline payload displays potent therapeutic activity in vivo. J. Control. Release.

[276] Tolcher AW (2021). A first-in-human study of mirzotamab clezutoclax as monotherapy and in combination with taxane therapy in relapsed/refractory solid tumors: Dose escalation results. J. Clin. Oncol..

